# Photodynamic treatment of pathogens

**DOI:** 10.1007/s40766-022-00031-4

**Published:** 2022-03-15

**Authors:** Pietro Delcanale, Stefania Abbruzzetti, Cristiano Viappiani

**Affiliations:** Dipartimento di Scienze Matematiche, Fisiche e Informatiche, Università degli Studi di Parma, Parco Area delle Scienze 7A, 43124 Parma, Italy

**Keywords:** Photodynamic effect, Singlet oxygen, Photosensitizer, Antimicrobial photodynamic treatment

## Abstract

The current viral pandemic has highlighted the compelling need for effective and versatile treatments, that can be quickly tuned to tackle new threats, and are robust against mutations. Development of such treatments is made even more urgent in view of the decreasing effectiveness of current antibiotics, that makes microbial infections the next emerging global threat. Photodynamic effect is one such method. It relies on physical processes proceeding from excited states of particular organic molecules, called photosensitizers, generated upon absorption of visible or near infrared light. The excited states of these molecules, tailored to undergo efficient intersystem crossing, interact with molecular oxygen and generate short lived reactive oxygen species (ROS), mostly singlet oxygen. These species are highly cytotoxic through non-specific oxidation reactions and constitute the basis of the treatment. In spite of the apparent simplicity of the principle, the method still has to face important challenges. For instance, the short lifetime of ROS means that the photosensitizer must reach the target within a few tens nanometers, which requires proper molecular engineering at the nanoscale level. Photoactive nanostructures thus engineered should ideally comprise a functionality that turns the system into a theranostic means, for instance, through introduction of fluorophores suitable for nanoscopy. We discuss the principles of the method and the current molecular strategies that have been and still are being explored in antimicrobial and antiviral photodynamic treatment.

## Introduction

The increasing occurrence of multidrug-resistant pathogenic microorganisms is becoming one of the major societal health challenges. It has been estimated that, in the absence of substantial increase in the rate of development of new effective drugs, the number of casualties related to these infections will increase from roughly 700,000 in 2016 up to nearly 10,000,000 in 2050 [1]. Microbial antibiotic resistance mostly arises from the excessive and inappropriate use of antibiotics for humans and for animals [2]. It has been estimated that in 2015, 42 billion daily doses of antibiotics have been administered for human use. Extrapolation of this figure to 2030 affords an estimate of roughly 128 billion daily doses [3]. Moreover, the amount of antibiotics employed with animals in 2014 was estimated to be nearly twice as large as that for human therapeutic purposes. [4] Importantly, antibiotics used to treat livestock are largely the same as those for humans. [5] In this scenario, the decreasing rate of development of new antibiotics faces the fast rising number of resistant bacteria [6, 7].

Thus, development of methods that are not prone to induction of resistance is of the greatest relevance. In this context, antimicrobial PhotoDynamic Inactivation (PDI) is emerging as an effective alternative strategy.

In essence, the photodynamic effect consists in the generation of reactive oxygen species (ROS) upon illumination of an otherwise inert molecular species, termed a photosensitizer (PS), by harmless visible light. These ROS selectively exert a cytotoxic action nearby their generation sites, i.e. against those cells where the PS is localized, and where light is shone. Given the generic action of the oxidative stress against a variety of molecular targets (lipids, proteins, nucleic acids, …) it is generally accepted that this approach is less subject to resistance [8]. The photodynamic approach was shown to lead to inactivation of multiresistant “ESKAPE” pathogens, drug-resistant bacteria strains such as vancomycin-resistant *Enterococcus faecium*, methicillin-resistant *Staphylococcus aureus* and the multi-drug-resistant species *Klebsiella pneumoniae*, *Acinetobacter baumannii*, *Pseudomonas aeruginosa* and *Enterobacter *ssp. [9]. Application of PDI has extended to virtually all types of pathogens, including bacteria [10–13], fungi [14–16], and viruses [17, 18].

The recently renewed interest in antimicrobial PDI has roots in the early twentieth century, with the first observation by O. Raab of photoinactivation of *Paramecium caudatum* when exposed to the dyes acridine or eosin and then to solar light. [19] This phenomenon was further investigated by von Tappeiner who confirmed that the deactivation of bacteria was not a consequence of heat, but rather a light-activated effect [20, 21]. The principle was applied to photoinduced tumor cell death by von Tappeiner shortly after [22]. Historical perspectives on antimicrobial PDI (sometimes also termed antimicrobial photodynamic therapy, aPDT) and the related photodynamic therapy of cancer (usually referred to as PDT) are available in the literature [23–25]. Although different acronyms are found in the literature, we will consistently use the term antimicrobial PDI in this review, when referring to the light induced photoinactivation of microorganisms treated with exogenous PS molecules. Unlike PDT of cancer, now a consolidate treatment in clinical practice [26], antimicrobial PDI is still in its infancy and its potential largely underappreciated.

This chapter is not intended to provide a comprehensive review of the field, rather to offer an overview of the physical–chemical principles and a selection of cases that demonstrate the potential of the method in the battle against pathogens, mostly bacteria and viruses. Several authoritative reviews on viral [18, 27–32] and bacterial [9, 13, 33–37] PDI have been published in recent years, to which we refer for comprehensive accounts on specific aspects. Although the clinical applications are far less developed than in cancer therapy, antimicrobial PDI is being clinically tested on localized infections [38, 39], including oral infections [40–42], and is expected to rapidly gain interest, in view of the current challenges related with the COVID-19 pandemic and the rising number of resistant bacterial strains.

It is worth remarking that the interest in antimicrobial PDI is driven also by other societal and environmental instances such as food safety [43] and the need to devise effective and environmentally friendly methods to treat microbial infections in agriculture [44].

## Photodynamic effect in essence

**Fig. 1:**
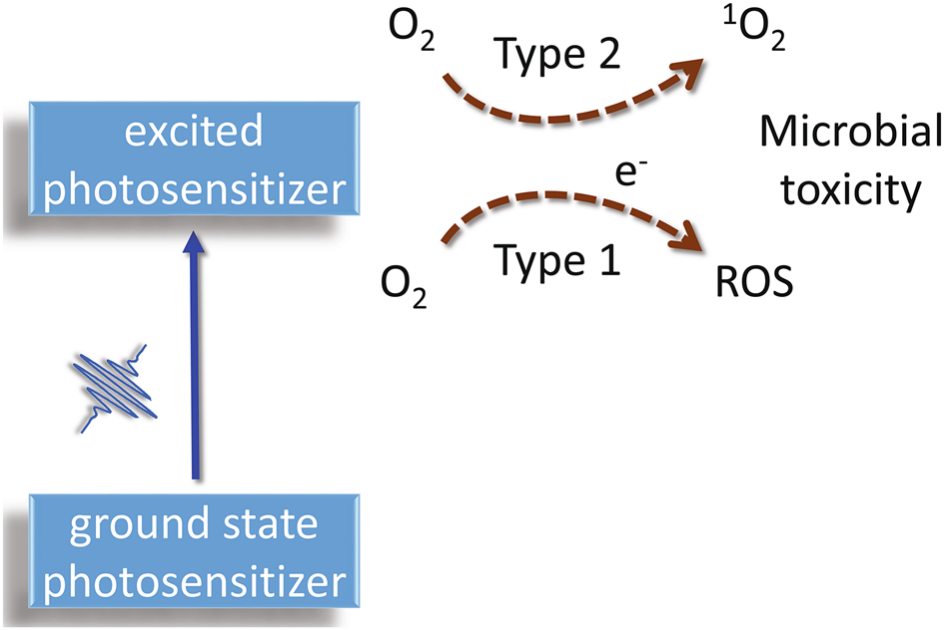
Schematic of the photodynamic effect

Over the years, it has been firmly established that the photodynamic effect is the consequence of the joined actions of three distinct elements—a molecule capable of absorbing light and generate ROS from its excited states, a light source, emitting at wavelengths where absorption from the molecule occurs, and molecular oxygen. When a photon is absorbed by the molecule, promotion to an electronically excited singlet state initiates a series of events, that includes radiative transitions such as fluorescence emission from the lowest electronically excited single state, and non-radiative relaxations (vide infra). For the photodynamic effect, the most relevant process is spin inversion in the excited state, a process that is forbidden by quantum mechanics, but is made possible thanks to spin–orbit coupling that becomes appreciable in several molecular systems, e.g. in cases when heavy atoms are present in the structure. As a consequence, a triplet state is formed, whose lifetime is long enough to allow energy and/or electron transfer reactions with molecular oxygen. These reactions lead to formation of the excited singlet state of molecular oxygen and/or superoxide anion. The triplet state of the PS may also undergo electron transfer reactions with biomolecules to generate other ROS. ROS then trigger a series of oxidative reactions that result in cellular toxicity (Fig. 1).

Reactions implying an electron transfer are usually termed Type 1 processes, whereas energy transfer to molecular oxygen is referred to as Type 2 process. Development of photoantimicrobial compounds generally favors photoactive molecules acting through Type 2 process, in view of the fact that bacterial defense strategies are aligned against oxidative stress in form of oxygen radicals [45–47].

**Fig. 2:**
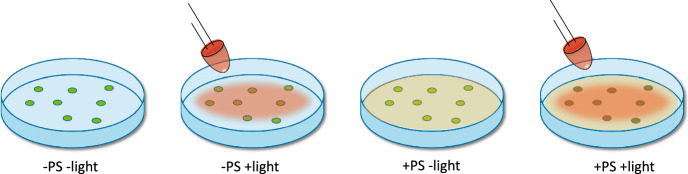
Viable bacteria (green dots) on a Petri dish (−PS −light) can be exposed to visible light (−PS +light) with no sizeable effects on their viability. Upon incubation with a suitable PS (+PS −light) no effects on viability are observed, and only when visible light of suitable wavelength is shone in the presence of PS a decrease in viability (brown dots) is observed (+PS +light)

Since the photodynamic effect is observed only when light of the proper wavelength, a PS, and oxygen are present simultaneously, photodynamic treatments are inherently local treatments which usually limits their application to specific infection sites. The method is intrinsically endowed with spatial and temporal selectivity, i.e. it exerts the cytotoxic action only when and where the PS, molecular oxygen and light are simultaneously present. Figure 2 illustrates this concept for bacteria on a Petri dish, upon incubation with a PS and exposure to visible light.

## The driving photophysics

**Fig. 3:**
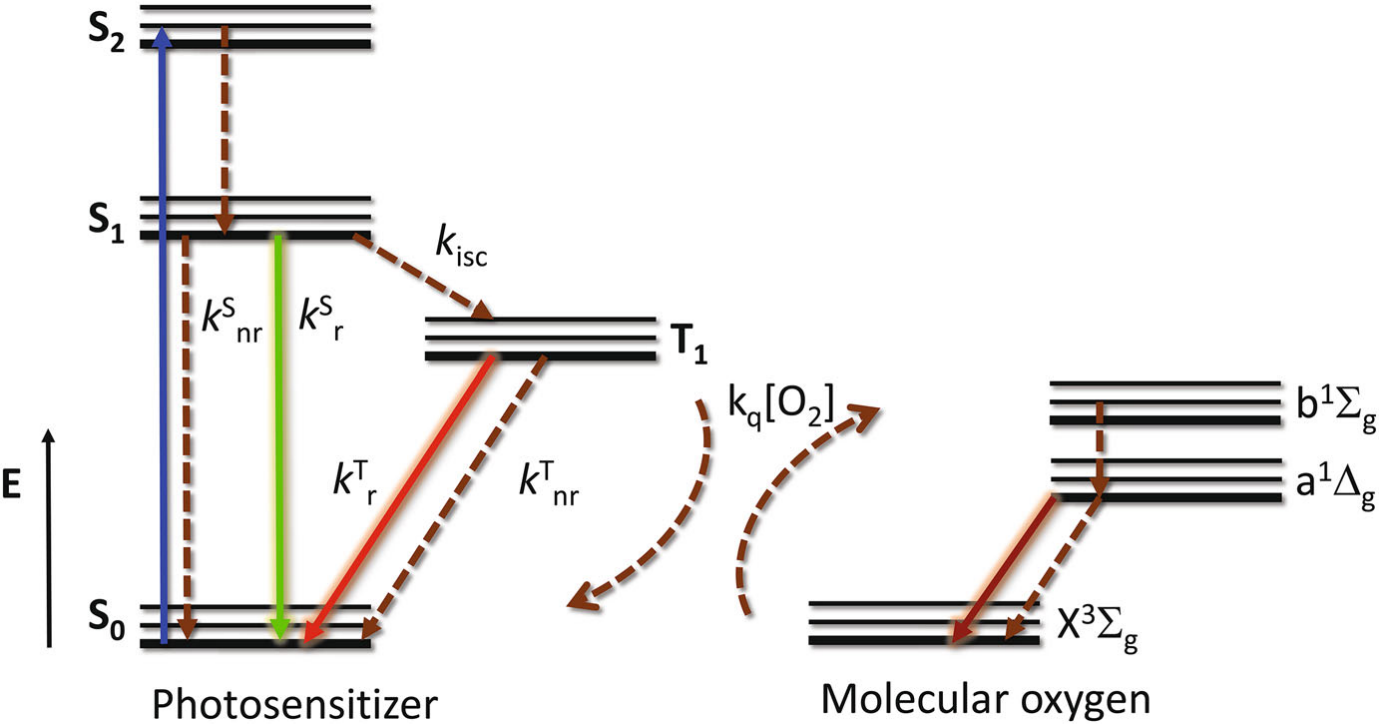
Simplified Jablonski diagram for a PS in the presence of molecular oxygen. The blue solid arrow indicates the electronic transition following photon absorption. Dashed arrows indicate non-radiative relaxations, solid arrows indicate radiative processes. Rate constants are reported next to relevant processes

The Jablonski diagram in Fig. 3 summarizes the main photophysical processes occurring after photo-excitation of a PS by a visible photon [48–50]. After absorption of light by the ground state S_0_ (blue arrow), an excited singlet state is populated, that rapidly (on the ps time scale) deactivates non-radiatively (brown dashed arrows) through vibrational relaxation and internal conversion to the lowest vibrational sublevel of the first electronically excited state (*S*_1_). The vibrational ground state within each electronic state is indicated by a thicker line. From this state, non-radiative relaxation (*S*_1_ → *S*_0_, brown dashed arrow, rate *k*^s^_nr_), radiative relaxation (*S*_1_ → *S*_0_, green solid arrow, termed fluorescence emission, rate *k*^s^_r_) or spin inversion (*S*_1_ → *T*_1_, brown dashed arrow, intersystem crossing, rate *k*_isc_) can occur on the nanosecond time scale. Triplet decay proceeds through non-radiative (*T*_1_ → *S*_0_, brown dashed arrow, intersystem crossing, rate *k*^T^_nr_) or radiative relaxation (*T*_1_ → *S*_0_, red solid arrow, termed phosphorescence emission, rate *k*^T^_r_), and is characterized by much longer relaxation times, due to the spin selection rule and the high energetic separation between *T*_1_ and *S*_0_ [48]. In the presence of molecular oxygen, an additional non-radiative relaxation pathway for the triplet state exists, due to a Dexter type, electron-exchange energy transfer, with a concentration dependent rate *k*_q_[O_2_]. This leads to either direct formation of either excited molecular oxygen O_2_(a^1^Δ_g_) or the higher energy state O_2_(b^1^Σ_g_) which, in solution phase systems, then decays rapidly and with unit efficiency to the O_2_(a^1^Δ_g_) state [51]. For simplicity singlet oxygen O_2_(a^1^Δ_g_) state will be indicated from now on as ^1^O_2_, when the use of the spectroscopic terms is not necessary. It is worth mentioning that, in addition to the mechanism outlined above, ^1^O_2_ may be produced upon oxygen quenching of the S_1_ state of a sensitizer. This process is generally associated with the *S*_1_–*T*_1_ intersystem crossing, and requires that the *S*_1_–*T*_1_ energy gap exceeds the ^1^O_2_ excitation energy [52]. In some cases, direct production of ^1^O_2_ upon *S*_1_–*S*_0_ de-excitation has been reported [53]. ^1^O_2_ may decay radiatively through phosphorescence emission (dark red solid arrow) or non-radiatively.

A relevant parameter for characterizing the ^1^O_2_ photosensitization efficiency of a photosensitizer is the ^1^O_2_ yield (*Φ*_Δ_), defined as the fraction of ^1^O_2_ molecules produced per absorbed photon (by the PS). Since ^1^O_2_ production implies two reaction steps (formation of the triplet state of the PS and energy transfer to molecular oxygen), *Φ*_Δ_ is the product of the triplet yield (*Φ*_T_ = *k*_isc_/(*k*^s^_nr_ + *k*^s^_r_ + *k*_isc_)) and the ^1^O_2_ efficiency (*η*_Δ_, the ratio between the number of sensitized ^1^O_2_ and the PS triplet states). Comprehensive compilations of *Φ*_Δ_ and ^1^O_2_ lifetime (*τ*_Δ_) values for a number of photosensitizers have been published in the 90’s [54–56].

**Fig. 4:**
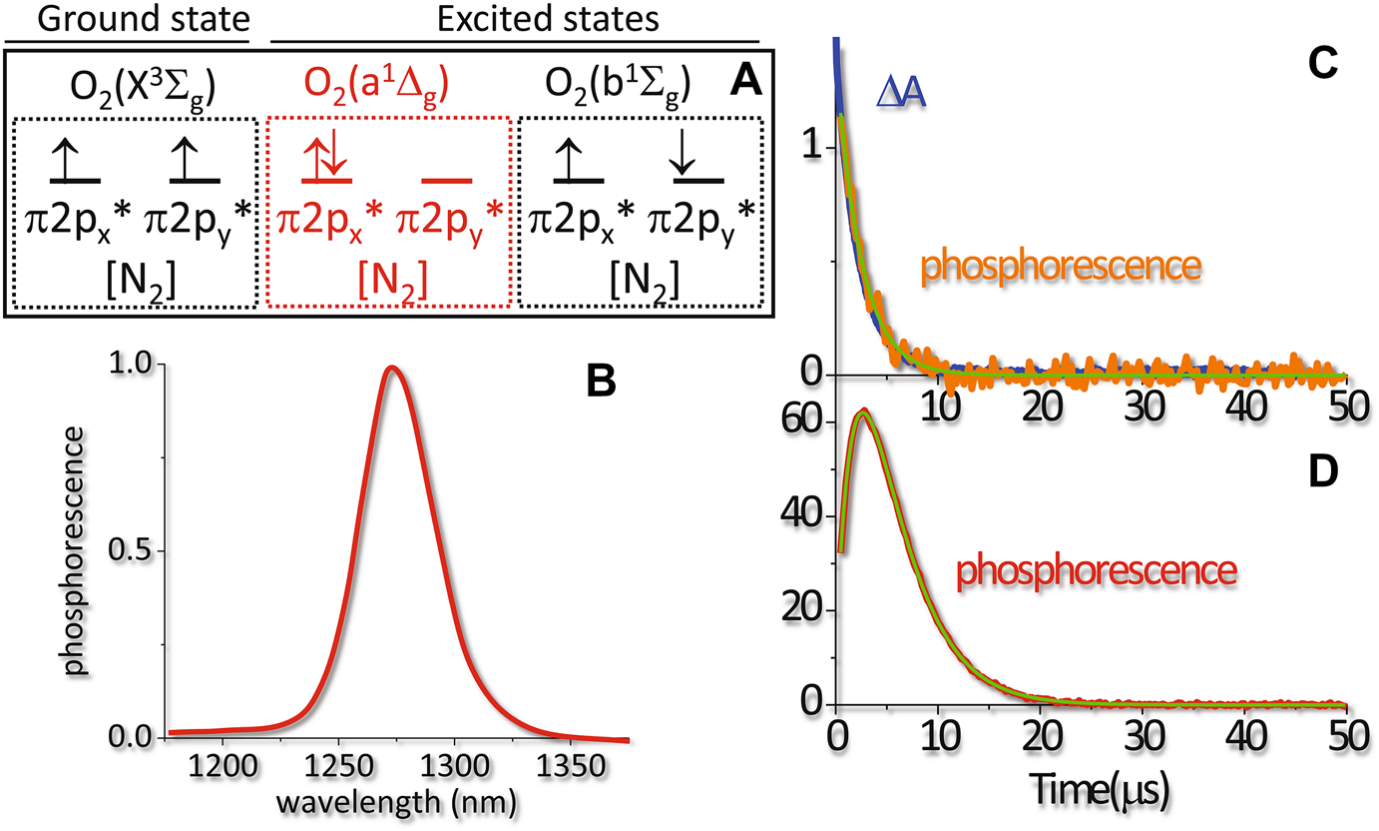
**a** Electronic configuration of molecular oxygen in the ground and in the excited states. **b** Near Infrared phosphorescence emission from ^1^O_2_ in water [63]. **c** Decay of the photosensitizer triplet state as monitored by nanosecond transient absorption (blue) and phosphorescence emission at 1100 nm (orange). The case reported is for Rose Bengal in air equilibrated PSB buffered solution at pH = 7.4. **d** Time resolved ^1^O_2_ phosphorescence at 1275 nm showing the typical rise and decay shape. The curve was collected for Rose Bengal in air equilibrated PSB buffered solution at pH = 7.4. Phosphorescence emission data are courtesy of prof. Santi Nonell (Institut Quimic de Sarria, Barcelona, Spain)

Molecular Orbital theory predicts that molecular oxygen contains a closed-shell structure identical to the one of molecular nitrogen plus two electrons located in π* antibonding orbitals (Fig. 4A). Since it is possible to combine the π* spatial wavefunctions and the spin wavefunctions in 6 spin-orbital wavefunctions antisymmetric to exchange of electrons, the electronic configuration of O_2_ gives rise to three state termed O_2_(X^3^Σ_g_), O_2_(a^1^Δ_g_), and O_2_(b^1^Σ_g_). According to Hund’s rule, the lowest energy configuration is O_2_(X^3^Σ_g_), with two unpaired electrons of total spin *S* = 1 and a triplet multiplicity, as confirmed by the paramagnetic properties of molecular oxygen ground state. The states O_2_(b^1^Σ_g_) and O_2_(a^1^Δ_g_) are located at 156.9 kJ/mol and 94.3 kJ/mol above the ground state O_2_(X^3^Σ_g_), respectively [51, 57].

In keeping with the above energetic levels, decay of molecular oxygen excited state is accompanied by phosphorescence emission from state O_2_(a^1^Δ_g_) that occurs in the near infrared, peaked at ~ 1275 nm (Fig. 4b) [58–62]. Decay of the PS triplet state (precursor of ^1^O_2_) can be monitored by triplet–triplet absorption (blue curve) or phosphorescence emission by the triplet state (orange) (Fig. 4c). The time profile of the two signals is identical as evidenced by the fitting curve (green). Time resolved ^1^O_2_ phosphorescence emission shows a typical rise and decay shape (Fig. 4d). For the reported case, the rise is due to energy transfer from the triplet state of the PS to the ground state molecular oxygen, leading to formation of O_2_(a^1^Δ_g_) state [58–62]. The decay reflects radiative (phosphorescence) deexcitation of O_2_(a^1^Δ_g_).

## It’s all about kinetics: time course of excited states population

**Fig. 5:**
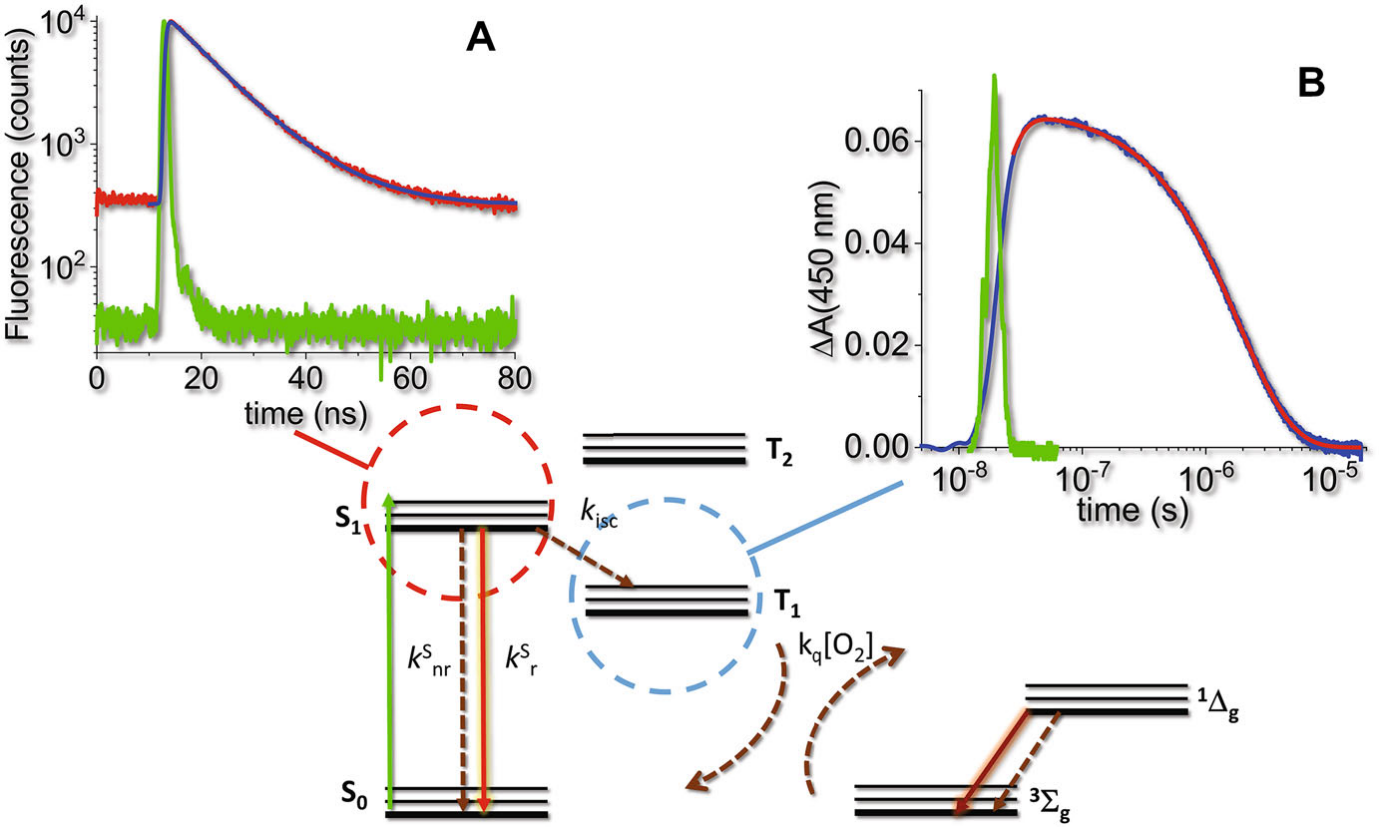
Photosensitizer triplet state formation and decay. Panel **a** shows the *S*_1_ state decay of a PS (4,4′,4′′,4′′′-(porphine-5,10,15,20-tetrayl)tetrakis(benzenesulfonic acid) tetrasodium salt hydrate, abbreviated in TSPP) measured through its red fluorescence emission (red curve) using time correlated single photon counting. The green curve is the instrument response function (IRF) for excitation with a sub-nanosecond pulsed LED. The blue curve is the fitting to a monoexponential decay using a reconvolution method [50]. Panel **b** reports the time profile for *T*_1_ formation (rising part observable in the nanoseconds) and decay (in the microseconds) through the triplet–triplet transient absorption of TSPP, as measured by nanosecond laser flash photolysis. The time axis is plotted on a log scale to appreciate both nano- and micro-second kinetics. The blue curve reports the triplet–triplet absorption changes after nanosecond excitation (green curve). The red curve is a fit to a biexponential relaxation. The lifetime of the rising part is identical to the *S*_1_ lifetime measured from TCSPC (panel **a**)

Formation of the triplet state T_1_ proceeds from the decay of the lowest energy electronically excited singlet state S_1_, therefore the time profile for decay of S_1_ through its fluorescence emission, occurring in the nanoseconds, reports also on the intersystem crossing kinetics (Fig. 5a). Accordingly, the same process can be followed in the rising part of the triplet–triplet transient absorption in nanosecond laser photolysis experiments (Fig. 5b), where the time evolution on longer time scales reflects triplet decay.

According to the Jablonski diagram (Fig. 2), decay of the triplet state of the PS after *δ*-pulse excitation is best described by the following rate equation, Eq. (1) [57]:

1 $$\frac{{{\text{d}}\left[ {T_{1} } \right]}}{{{\text{d}}t}} = - \left( {k_{{\text{r}}}^{{\text{T}}} + k_{{{\text{nr}}}}^{{\text{T}}} + k_{{\text{q}}} \left[ {{\text{O}}_{2} } \right]} \right)\left[ {T_{1} } \right],$$

where $\tau_{{\text{T}}}^{{0}} = \frac{1}{{k_{{\text{r}}}^{{\text{T}}} + k_{{{\text{nr}}}}^{{\text{T}}} }}$ is the intrinsic triplet lifetime of the PS in the absence of any quencher. *τ*^0^_T_ usually falls in the 10^–4^–10^–3^ s range [64]. Integration of this differential equation with the initial condition [*T*_1_](0) = [*T*_1_]_0_ yields the time course for triplet state decay, Eq. (2):

2 $$\left[ {T_{1} } \right] = \left[ {T_{1} } \right]_{0} {\text{e}}^{{ - \left( {\frac{1}{{\tau_{{\text{T}}} }} + k_{{\text{q}}} \left[ {{\text{O}}_{2} } \right]} \right)t}} .$$

Since in most cases, *1/τ*^0^_T_ ~ 10^3^–10^4^ s^−1^ and *k*_q_ ~ 10^9^ M^−1^ s^−1^, for air equilibrated aqueous solutions the relatively high oxygen concentration ([O_2_] ~ 200 μM) almost entirely determines the overall rate constant for the triplet decay. Thus, the observed lifetime of triplet states (*τ*_T_) in air equilibrated aqueous solutions is ~ 2 μs for most triplet states. The higher solubility of molecular oxygen in organic solvents results in lower triplet lifetimes. The above decay can be followed by either triplet–triplet time resolved absorption (by nanosecond laser flash photolysis, see e.g. the blue curve in Figs. 4c, 5b) or by time resolved phosphorescence emission (see e.g. the orange curve in Fig. 4c).

**Fig. 6:**
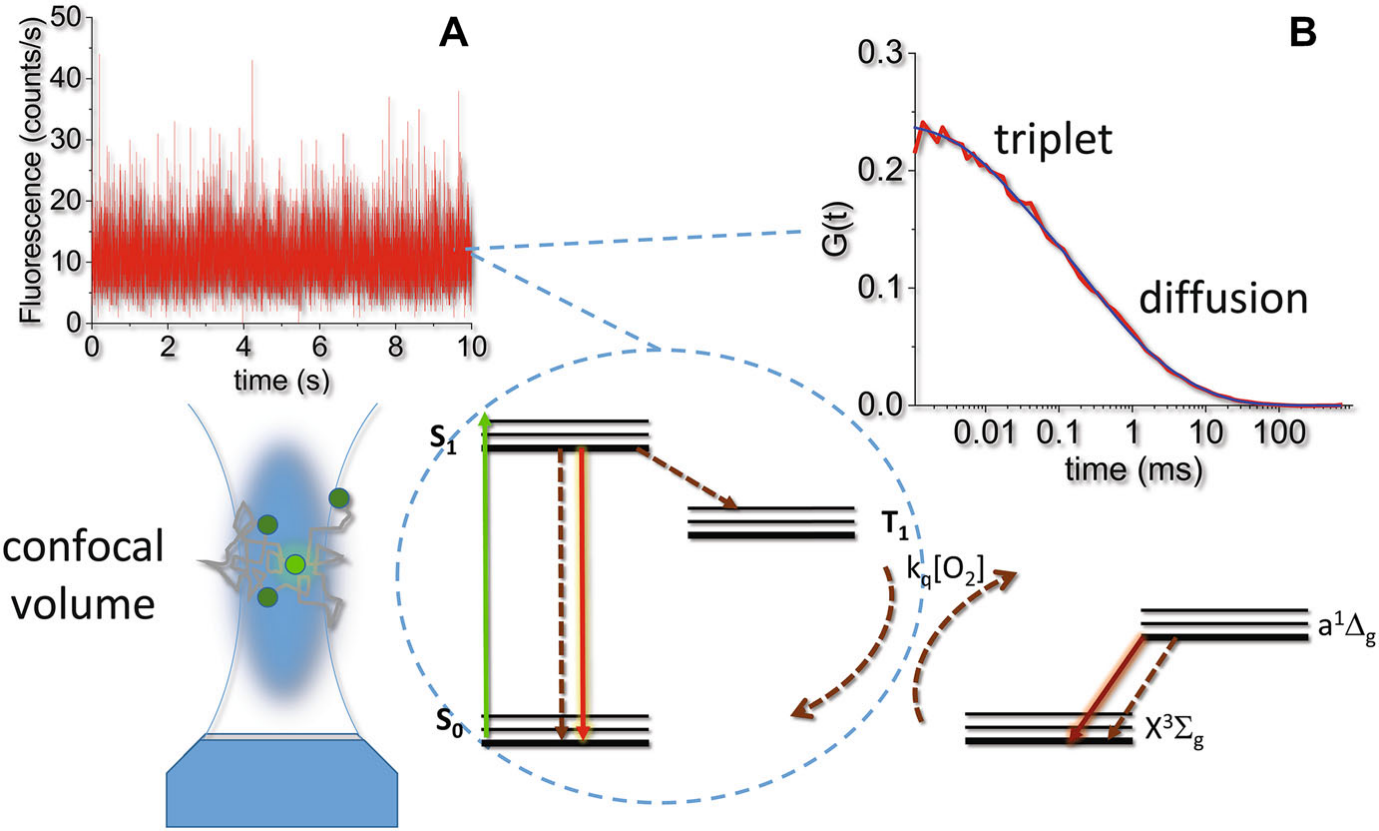
Schematic representation of the concepts behind fluorescence correlation spectroscopy. A fluorescence molecule inside the detection volume of a confocal microscope is excited giving rise to green emission, resulting in a high fluorescence signal (panel **a**). When the molecule exits the confocal volume, a decrease in fluorescence intensity is observed, which is recovered as another molecule enters the confocal volume. These fluctuations reflect diffusion of the fluorophore. A further source of fluctuations on shorter time scales is the photoinduced formation of dark states (as triplet states) prior to exit the confocal volume. Panel **b** reports the autocorrelation curve for the signal in panel **a**. The time scales corresponding to molecular diffusion and triplet state kinetics are indicated. The experimental data are taken on a complex between bovine serum albumin and Hyp (~ 10 nM). Excitation was at 475 nm and detection at 650 nm [68]

Photoinduced formation of the PS triplet state can be detected also using fluorescence correlation spectroscopy, an equilibrium fluctuation method with single molecule sensitivity [65–67]. Fluctuations may arise from diffusion of the fluorescent molecules that enter and exit the detection volume in a confocal microscope (Fig. 6a). The autocorrelation function of the time trace allows to estimate the diffusion coefficient of the diffusing fluorescent species (Fig. 6b). Of interest to the photophysics of the molecule under investigation, on the short micro-second time scale, it is possible to detect fluctuations arising from formation and decay of the triplet state of the PS, a dark state that is populated with the molecule still inside the confocal volume. The extent of triplet state formation and it decay kinetics allow to demonstrate formation of triplet states also for very dilute samples, which would be difficult, if not impossible, to measure with nanosecond laser flash photolysis. This is quite important, e.g., for samples at the development stage when available amounts are sometimes minute.

The autocorrelation function for a single diffusing species with diffusion coefficient *D*, undergoing intersystem crossing to a triplet state, can be written as [66]:

3 $$g\left( t \right) = \frac{1}{N}\frac{{1 - \Theta_{{\text{T}}} + \Theta_{{\text{T}}} {\text{e}}^{{ - \frac{t}{{\tau_{{\text{T}}} }}}} }}{{1 - \Theta_{{\text{T}}} }}\left( {1 + \frac{t}{{\tau_{{\text{D}}} }}} \right)^{ - 1} \left( {1 + \frac{t}{{\gamma^{2} \tau_{{\text{D}}} }}} \right)^{ - 1/2} ,$$

where *τ*_D_ is the diffusion time, *N* is the average number of particles in the confocal volume, $\gamma = \frac{{z_{0} }}{{w_{xy} }}$ is the ratio between the major ellipsoid axis (along the optical axis) and *w*_*xy*_ is the ellipsoid radius in the focal plane. Finally, the following relation holds: $D = \frac{{w_{xy}^{2} }}{{4\tau_{D} }}$, that connects *τ*_D_ and the diffusion coefficient. The term *Θ*_T_ represents the amplitude of the triplet state signal, whereas *τ*_T_ is the triplet lifetime.

Photosensitized ^1^O_2_ production is conveniently followed by its near infrared (NIR) phosphorescence emission. In a typical time resolved experiment, formation of the NIR phosphorescence emission (see red curve in Fig. 4d) occurs with the same rate of triplet decay (1/*τ*_T_). Decay of the phosphorescence signal occurs with lifetime *τ*_Δ_, which in neat water is 3.3 μs. The overall time course is described by Eq. (4) [63, 69]. Note that if *τ*_T_ > τ_Δ_, then the signal rises with lifetime τ_Δ_ and decays with lifetime *τ*_T_.

4 $$S = S_{0} \frac{{\tau_{\Delta } }}{{\tau_{\Delta } - \tau_{{\text{T}}} }}\left( {{\text{e}}^{{ - \frac{t}{{\tau_{\Delta } }}}} - {\text{e}}^{{ - \frac{t}{{\tau_{{\text{T}}} }}}} } \right)$$

Besides allowing determination of precise estimates of *Φ*_Δ_ and *τ*_Δ_, time resolved near infrared phosphorescence emission provides a tool to identify Type 2 (^1^O_2_ formation by electron-exchange energy transfer) photosensitization processes. The kinetics can be selectively influenced by physical chemical conditions with known consequences on *Φ*_Δ_ and *τ*_Δ_.

Perhaps the most striking effect, is that singlet oxygen lifetime can be affected by perturbing the excited state through the H_2_O/D_2_O solvent isotope effect. The lifetime of ^1^O_2_ in D_2_O (about 67 μs) is substantially longer than that in H_2_O (3.5 μs) [56]. The isotope effect reflects the coupling of ^1^O_2_ with high frequency OH vibrational modes of the solvent through non-radiative relaxations [51]. Tampering with the de-excitation rate constant, reflecting physical interaction with the solvent, provides a tool to assess the kinetic competition with other reactive de-excitation pathways. In the same line, adding molecules known to specifically quench ^1^O_2_ provides a similar kinetic information on competitive reaction pathways [52].

The near infrared phosphorescence emission accompanying the O_2_(a^1^Δ_g_) → O_2_(X^3^Σ_g_) transition is the most specific probe that enables to detect ^1^O_2_ photosensitization. Therefore, time resolved near infrared phosphorescence emission is the method of choice to demonstrate and quantitate ^1^O_2_ formation [63]. Nevertheless, the weakness of this spectroscopic signal poses limitations to its use in some applications, especially when it comes to cellular studies, where the use of long acquisition times and incubation of cells in deuterated buffers have been proposed to overcome the signal intensity issues [70, 71].

The use of fluorescent chemical traps for ^1^O_2_ provides an interesting alternative. One such probe is Singlet Oxygen Sensor Green^®^ (SOSG), a cell-impermeant, commercially available fluorescent sensor for ^1^O_2_. SOSG has been successfully applied to the detection and imaging of ^1^O_2_ photosensitization in plant leaves as a defense mechanism [72] and photoinactivation of *Staphylococcus aureus* bacteria [73].

SOSG is weakly fluorescent in the blue under near UV excitation. In the presence of ^1^O_2_, SOSG emits green fluorescence with excitation and emission maxima at 504 and 525 nm, respectively. The green fluorescence emission was assigned to an endoperoxide generated by the interaction of ^1^O_2_ with the anthracene component of SOSG [74]. It should be mentioned that, in spite of the usefulness as a ^1^O_2_ sensor, SOSG was shown to photosensitize ^1^O_2_, particularly under UV radiation. Moreover, under these conditions photobleaching is observed, due to the formation of radical species [75]. It was also reported that the probe is not suitable for use in the presence of ionizing radiation [76].

The properties of ^1^O_2_ fluorescent probes have been discussed [77].

## As long as life endures: sphere of action of singlet oxygen

It has been emphasized that the photodynamic effect has a double spatial (temporal) selectivity, as it exerts the cytotoxic action only where (when) the PS is uptaken by the target cell and where (when) light is shone. When ^1^O_2_ is produced inside or close to a cell, its activity will be limited to the environment in the immediate vicinity of this location, the size of this volume depending on the diffusion of the excited molecular species during its lifetime [78].

When it comes to antimicrobial applications of the method, the meaning of localization is stringent, as it becomes clear in the following. When ^1^O_2_ is produced at the location where the PS has been excited, it will diffuse through the surrounding medium. The distance d traveled by ^1^O_2_ depends on the diffusion coefficient D and can be approximately expressed as *d* ~ $\sqrt {6Dt}$ [79]. The time span relevant for diffusion is related to the ^1^O_2_ lifetime τ_Δ_. Decay of the ^1^O_2_ population is roughly complete after a time *t* ~ 5*τ*_Δ_, and this implies that the maximum distance sampled by ^1^O_2_ before decaying is about *d* ~ $\sqrt {30D\tau_{\Delta } }$ [52].

**Fig. 7:**
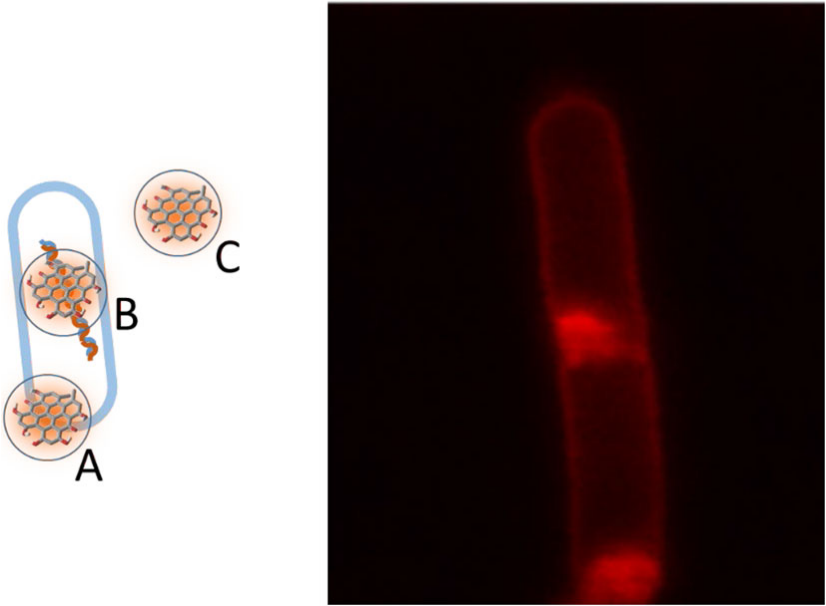
Left. The photosensitizing molecule should be in close vicinity (~ 200 nm, indicated by the light blue circles, not to scale) of sensitive molecular components such as wall membranes or proteins (position A) or cytoplasmic components such as nucleic acids (position B). If the PS is in the extracellular space at distance exceeding 200 nm (position C) no cytotoxic effects will be observed. Right. STED nanoscopy of Hyp bound to *E. coli*. The PS is localized on the wall, associated with the membrane. STED image by Dr. Paolo Bianchini, Istituto Italiano di Tecnologia, Genova, Italy

Considering that the lifetime of ^1^O_2_ in water is about 3.3 μs [51, 56, 63, 80] and that the diffusion coefficient for O_2_ at 20 °C is *D* = 1800 μm^2^/s [81], the maximum distance traveled by ^1^O_2_ in water is around 400 nm. The effect of the cellular environment on ^1^O_2_ lifetime has been studied for a reported lifetime of ≈ 3 μs which for a *D* ~ 400 μm^2^/s, corresponds to a diffusion length of about 200 nm [80]. This sets an upper limit to the distance between the PS and the biological target molecules for the photosensitized species to have cytotoxic effects. Thus, PSs must be brought in close vicinity of the cellular structures to be perturbed or destroyed, as indicated in Fig. 7.

It should be emphasized that cell environments are micro-heterogeneous and are expected to result in different values of *D* and *τ*_Δ_ in different compartments, so the above estimate of the sphere of action (200 nm) should be considered an average upper limit [51, 70].

The development of instrumentation capable of monitoring the time profile of ^1^O_2_ formation and decay through its phosphorescence emission at ~ 1275 nm has allowed an extensive characterization of the kinetics for formation and decay of this reactive intermediate under a variety of conditions in solution phase [51], and it was found that rate constant for ^1^O_2_ decay is sensitive to interacting molecules present in solution [56].

The rate constant for ^1^O_2_ decay *k*_Δ_ = 1/*τ*_Δ_ receives contributions from all processes leading to removal of the species. It was proposed that *k*_Δ_ is described by the following expression [52]:

4 $$k_{\Delta } = k_{{{\text{nr}}}} \left[ {S_{1} } \right] + k_{{\text{r}}} \left[ {S_{1} } \right] + \sum\limits_{i} {k_{qi} \left[ {Q_{i} } \right]} + \sum\limits_{i} {k_{rxni} \left[ {R_{i} } \right]} ,$$

where the first two terms are due to the interaction with the solvent and are described by pseudo-first-order rate constants times the concentration of the solvent for the non-radiative (*k*_nr_) and radiative (*k*_r_) decays, respectively. In the case of water [*S*_1_] would be ~ 55 M. In neat water the lifetime of ^1^Δ_g_ is 3.5 μs [56] whereas in D_2_O the lifetime increases to 67 μs [82].

It is important to observe that the phosphorescence emission from ^1^O_2_ is very weak since in water *k*_r_/*k*_nr_ ~ 10^–6^. A remarkable increase in sensitivity is obtained if D_2_O is used instead of H_2_O, since *k*_r_[H_2_O] ~ *k*_r_[D_2_O] and *k*_nr_[H_2_O] ~ 20*k*_nr_[D_2_O], which results in higher phosphorescence emission [51, 62]. In micro-heterogeneous media such as in a cellular environment, the solvent composition shows spatial variations leading to changes in phosphorescence lifetime and yield, and the first two terms are conveniently replaced by summations over several solvents.

The presence of physical quenchers (*Q*_*i*_) or chemically reactive species (*R*_*i*_) adds up in the above expression, contributing to the overall rate constant for ^1^O_2_ decay [56].

Early estimates of ^1^O_2_ lifetime in cells, based on indirect methods, afforded a value of 10–300 ns, an indication of strong quenching of ^1^O_2_ by the cellular environment [83]. However, the estimate has been recently reconsidered and a value of 3.0 μs was proposed [70, 84, 85], not far from the value in water (3.5 μs). This was more recently confirmed in independent experiments [80].

These findings suggest that although cellular environments are rich in potentially reactive functional groups capable of removing ^1^O_2_, their effective concentration/availability may be reduced to an extent that the main determinant of ^1^O_2_ decay remains the physical interaction with the solvent. For instance, although several amino acids in denatured proteins are good quenchers of ^1^O_2_, in a native protein they may be buried in the protein structure and thus become inefficient [86]. The smaller than expected contribution of physical quenching and chemical reactivity from cell components was further suggested by studies showing that cell death mediated by ^1^O_2_ depended on the D_2_O/H_2_O ratio in the surrounding medium [87]. This evidence hints to a kinetic competition between solvent-dependent ^1^O_2_ deactivation channels and chemical processes leading to phototoxicity [88–90].

The overall result of kinetic studies aimed at elucidating rate constants for deactivation of ^1^O_2_ though chemical reactions is that these rates generally fall in the range 10^5^–10^8^ M^−1^s^−1^, and only a few molecules remove ^1^O_2_ with rate constants that approach those characteristic of the diffusion-controlled limit (i.e., 10^9^–10^10^ M^−1^s^−1^) [51] [56].

It is worth mentioning that in both single-cell and ensemble experiments, in some cases, a change in the kinetics of the ^1^O_2_ phosphorescence as a function of the elapsed photolysis time may be observed. Both formation and decay of the near infrared emission progressively slow down as the photolysis time is increased, a fact that has been interpreted as reflecting local changes in cell environment, associated with the extensive photosensitization [70, 91].

Effects of prolonged illumination may also show up in the photophysical properties of nanostructured PSs as in the case of miniSOG, a flavin-binding protein that had been developed as a genetically encodable PS [92].

**Fig. 8:**
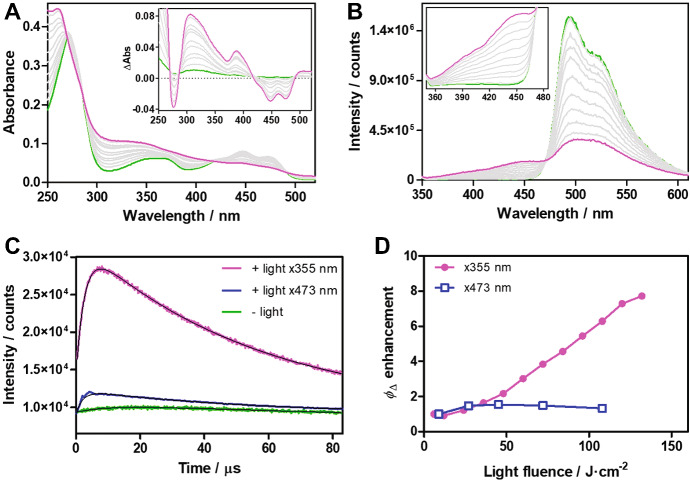
Spectral changes upon progressive illumination of miniSOG. **a** Changes in absorption spectra (difference spectra are reported in the inset). **b** Changes in fluorescence emission spectra. The presence of clear isosbestic points demonstrates the photoconversion between two species. Insets show the difference spectra between irradiated and non-irradiated samples. **c** Time-resolved NIR phosphorescence decays of native (green) and photoconverted miniSOG excited at 473 nm (blue) or 355 nm (magenta). **d** Observed *Φ*_Δ_ enhancement at 473 nm (blue) and 355 nm (magenta). Figure reproduced from Torra et al. [94]

Under low level of illumination, miniSOG generates ^1^O_2_ in low yield, because of a combination of limited oxygen accessibility and quenching of the FMN chromophore by electron-rich side chains, as observed for other flavin binding photoreceptors [93]. Upon prolonged, or intense irradiation with blue light several photoinduced changes are observed, that include photodegradation of flavin mononucleotide (FMN) into lumichrome and oxidation of the side chains of several amino acids that are responsible for quenching of the FMN triplet state [94]. As a consequence, dramatic spectral changes (both in the optical absorption and the fluorescence emission spectra) are observed (Fig. 8) and the ^1^O_2_ quantum yield increases substantially for the photoconverted miniSOG. Careful comparison between the crystal structures of dark- and photoconverted miniSOG shows that in the latter structure, molecular oxygen can more readily access the FMN. At the same time, oxidized electron-rich side chains cannot quench the triplet state of the chromophore.

## An overview on antimicrobial photosensitizers

**Fig. 9:**
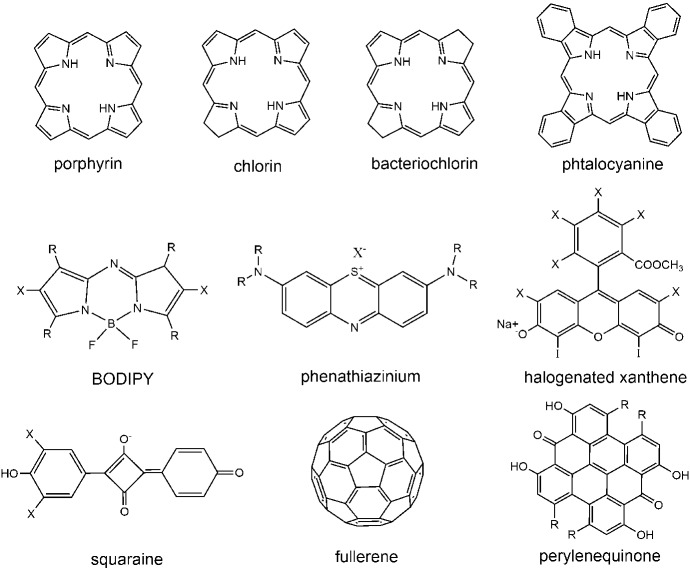
Representative structures for PS families used in antimicrobial PDI

Recent surveys of suitable photoantimicrobials for PDI of microorganisms have summarized their properties [18, 37, 95–97]. Several classes of compounds have been proposed and tested as photoactive antimicrobials, either driven by existing structures or by rational design. Representative structures for the main families of PS molecules discussed in this Section are reported in Fig. 9. Some of these compounds are of natural origin like curcumin, riboflavin, hypericin (Hyp) and psoralens. Photosensitization by tetrapyrrole macrocyclic compounds has been known for many years, especially in connection with cancer therapies. These compounds comprise porphyrins, chlorins, phthalocyanines and bacteriochlorins, for which the lowest photon absorption energy moves toward the near infrared region of the electromagnetic spectrum along the series. An additional class of PSs that are in use includes phenothiazinium dyes, such as methylene blue and toluidine blue. Several non-phenothiazinium dyes with xanthene, triarylmethane and indocyanine structures, as well as new structures based on BODIPY, squaraine and fullerene cages have been proposed. Selected examples of applications from an existing vast literature are reported in this section.

**Fig. 10:**
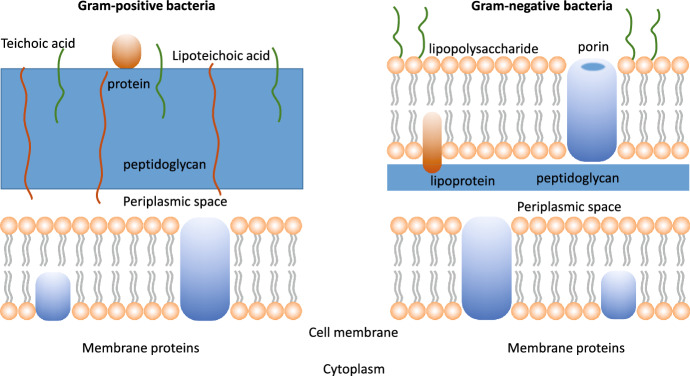
Pictorial representation of Gram-positive (left) and Gram-negative (right) cell walls. [239, 242]

In general, photoinactivation of pathogens has proven more effective on Gram-positive bacteria due to their single-membrane cell wall structure that allows deeper penetration of the PS (Fig. 10). Photoinactivation of Gram-positive bacteria is readily obtained with neutral or mono-cationic PSs. The well-organized and thick outer membrane in Gram-negative bacteria limits the penetration of the PS through the cell wall and makes these bacteria more resistant to PDI [98]. Positively charged PS molecules have been reported to be more effective. Modification of the PS molecules with the introduction of specific structures for targeting purposes will be discussed in Sect. 8.

### Porphyrin-related structures

Porphyrins are the best known family of PSs, that contains hematoporphyrin derivatives, an ensamble of hematoporphyrin monomers and oligomers of different size [99], and its purified version Photofrin^®^. In the’90 s, Photofrin^®^ has obtained the approval for the use in PDT against esophagus, lung and bladder tumors [100], but its efficacy in killing antibiotic-resistant bacteria has been also widely proven [101, 102]. Moreover, a very large number of modified porphyrins has been tested and some of them appear to be very promising agents with a broad spectrum of antibacterial activity (methicillin-resistant and methicillin-sensitive *S. aureus*, methicillin-resistant *S. epidermidis* and *E. coli*), like XF70 [103], a bis-cationic porphyrin which is effective also in the dark [104, 105]. This class of cationic porphyrins, in addition to tetra-substituted *N*methyl-pyridyl-porphine [106] and tri-meso(*N*methyl-pyridyl), meso (*N*-tetradecyl-pyridyl)porphine [107] have also demonstrated to be able to help in eradicating biofilms.

The capability of this class of molecules to photo-kill viruses has been reported [29, 108]. Recently, synthetic porphyrins have received particular attention, mainly cationic porphyrin derivatives [109, 110], *N*-methylpyridyl-substituted compounds [111] and complexes of 5,10,15,20-tetrakis(4-sulfonatophenyl) porphyrin with different metals [112], for the effectiveness on HIV. Interestingly, the problem of induction of resistance upon photo-inactivation of viruses has been discussed [113].

5-Aminolevulinic acid (ALA) is related to this class of molecules, although its structure does not contain a tetrapyrrole ring, because ALA is a precursor of a cellular endogenous PS, protoporphyrin IX. The administration of ALA induces protoporphyrin IX synthesis in all nucleated cells, thus it can be used for bacteria, fungi, and parasites eradication. Applications of the method have been reviewed [114]. It is worth to remember that ALA is extensively used in dermatology for dealing with skin lesions, either neoplastic or not [114], including viral lesions [115].

Moreover, ALA, PPIX and its derivatives, methyl aminolevulinate and hexaaminolevulinate [116, 117], as well as haematoporphyrin derivatives, have been widely exploited against viruses, demonstrating their efficacy [118, 119]. Perhaps the most important application of these molecules is in the treatment of HPV infections [120].

Importantly, the presence of endogenous photosensitizing porphyrins in pathogenic microbes, capable of generating ROS upon blue light illumination, has been exploited to achieve antimicrobial blue light inactivation without the need to apply exogenous PS molecules. The methodology was covered in recently published reviews [121, 122].

### Chlorins

The weak absorption exhibited in the red portion of the visible spectrum and the relatively slow metabolism process from tissues for porphyrin-related molecules, causing weeks-long photosensitivity [123], represent the main disadvantages of porphyrins. To overcome these problems and to confer a larger tissue penetrability to PDI, attention has been paid to two classes of PSs, obtained by reducing one (chlorin) or two (bacteriochlorin) pyrrole double bonds and thus moving the absorbance maximum to 650–670 nm and 730–800 nm, respectively. To enhance affinity for bacterial structures, a cationic polymer was conjugated to chlorin(e6) [124, 125]. The efficacy of this methodology has been demonstrated in a mouse model of burn infection [126].

### Bacteriochlorin derivatives

Although this class of PSs shows intriguing potentialities thanks to its far red absorption, problems may arise from oxidation processes they may accidentally undergo. In this respect, amphiphilic bacteriochlorins, obtained through synthetic or semi-synthetic pathways, appear particularly convenient [127], because depending on the inserted substitution, they show specific antimicrobial activity against Gram-positive bacteria (the di-quaternized bacteriochlorin), or Gram-negative bacteria (the tetra-quaternized and hexa-quaternized analog) [128]. Moreover, the action against viruses is well established [129], in particular for some derivatives. For example, 3-phorbinepropanol-9,14- diethyl-4,8,13,18-tetramethyl-20-(3*S-trans*) [130] has demonstrated its efficacy to photo-kill both enveloped and not enveloped viruses [131]. Interestingly, Temoporfin appeared to produce significant inactivation of Zika virus in the dark [132].

### Phthalocyanines

Phthalocyanines are interesting molecules because they combine an absorption maximum in the far red (670–780 nm) with a relevant molar extinction coefficient (larger than 10^5^ M^−1^cm^−1^). The macrocyclic ring can coordinate different atoms, and the most commonly used phthalocyanines contain zinc, aluminum and silicon. It is worth to note that the addition of cationic groups makes these molecules not only soluble, but also effective against Gram-negative bacteria, as observed against *E. coli* [133] *Aeromonas hydrophila* [134], methicillin-resistant *S. aureus*, *E. faecalis*, *P. aeruginosa* [135], when used together with Ca^2+^ and Mg^2+^ ions, in a “self-promoted uptake pathway” [133]. Phthalocyanines can also be modified to selectively bind bacteria, with the aim to design a targeted agent. An example of this approach has been proposed by Galstyan et al. [136], that synthesized a maltohexaose-conjugated Si(IV)-phtalocyanine able to completely kill methicillin-resistant *S. aureus* and decorate the outer membrane of both Gram-positive and negative bacteria, and a mannose-conjugate that conversely photoinactivates and labels only Gram-positive strains. The usefulness of phthalocyanines against viral infection in blood is well established since the 90 s [137] and has been recently reviewed [29]. In general, phthalocyanines appear to be significantly effective only on enveloped viruses [138].

### Other cyclic polypyrrole structures

In this class of molecules, that contains pyrrole rings variably inserted into macrocycles, porphycene, a structural isomer of porphyrin, is the most important system to be considered. In fact, the red absorption bands can be tuned between 620 and 760 nm [139] and a tricationic variant, named Py3MeO-TBPo, shows a broad spectrum effectiveness against both Gram-positive and negative bacteria, and potential applications in vivo as demonstrated on a methicillin-resistant *S. aureus* mouse infection model [140]. Very recently, conjugation of porphycene TMPo with an antibiotic, like gentamicin, has proposed a promising strategy to increase the antimicrobial activity of the two single components in vitro against both Gram-positive and Gram-negative bacteria (*S. aureus* and *E. coli*, respectively) [141].

### Natural products and derivatives

#### Curcumin

Curcumin is a natural chromophore extracted from *Curcuma longa* and it is well known for its therapeutic properties [142, 143]. Its efficacy in killing *S. mutants* for dental applications using blue LED light has been widely demonstrated not only in vitro [144–146], but also in vivo through a randomized clinical trial [147]. Moreover, modified asymmetric and glycosylated curcuminoids have been developed and tested in their photoinactivation potential against Gram-positive and negative strains [148]. To overcome poor hydrophilicity and consequent low bioavailability, several chemical modifications of curcumin carrying positive charges have been developed [149–152]. Effective treatment with curcumin was demonstrated for a wide spectrum of viruses, comprising influenza A virus [153]. Bioconjugates with peptides and fatty acids, synthetized to improve the lipophilicity, allowed photo-killing of vesicular stomatitis virus, feline corona and herpes viruses [154]. Interestingly, curcumin appears to have different targets in cells infection. As an example, its efficacy was proved against norovirus surrogates, inducing damages to nucleic acids and capsid proteins [155], hepatitis B [156] and C [157], and CoxB3 [158]. Moreover, curcumin proved useful in the treatment of HIV [159–161]

#### Riboflavin

Riboflavin is better known as vitamin B2 and is necessary for many reactions catalyzed by flavoproteins as part of the functional group of FAD and FMN cofactors.

It has demonstrated to be able to photo-inactivate Gram-positive bacteria *S. aureus*, *P. aeruginosa* [162], and *B. subtilis* [163] upon illumination with blue light, exploiting its absorption peak at 440 nm. Recently, riboflavin derivatives substituted with up to eight positive charges showed considerable improvement in photodynamic efficacy [164]. Importantly, riboflavin is one of the three PSs approved for clinical application in blood decontamination [165]. It is able to inactivate both enveloped and not enveloped viruses, as HIV, West Nile, VSV, IAV, pseudorabies virus, human hepatitis A virus, encephalomyocarditis virus, Sindbis virus, and MERS coronavirus [166]. Pathogens inactivation is due to damages to nucleic acids produced by hydrogen peroxide and hydroxyl radicals produced by oxidation of guanine bases [167].

#### Perylenequinones (hypericin, hypocrellin)

This class collects red absorbing pigments with a pentacyclic conjugated chromophore [168]. Hyp, extracted from *Hypericum perforatum*, is its most important component and has demonstrated a relevant antimicrobial activity both as purified molecule and in extracts from the plant [169–171]. However, even if it is a potent photodynamic agent against Gram-positive bacteria, the absence of charges on the chromophore makes Hyp ineffective for Gram-negative strains. To overcome this limitation, the use of electroporation has been suggested [172] and two cationic derivatives have been synthetized including the quaternary *N*,*N*,*N*-trimethyl-anilinium derivative [173]. Hyp was applied in food decontamination from pathogens [174, 175].

Other two interesting perylenequinones are two fungal metabolites, hypocrellin and cercosporin. The former received attention because it is able to kill Gram-positive bacteria [176, 177], the latter because its photodynamic activity occurs through the peroxidation of the cellular membrane [178]. The antiviral action appears very diversified in vitro, and activated not only by light but also in the dark [179].It is worth noting that treatments in vivo (clinical trials at the end of ‘90 s on patients with hepatitis C virus and HIV) were not satisfying [180, 181].

#### Psoralens

These compounds belong to the furanocoumarin family, obtained from coumarin by adding a furan ring, and are present in a number of fruits and vegetables, for example, figs, celery, carrots, parsley. Unlike most PSs, the mechanism underlying their phototoxic activity is independent of molecular oxygen. Psoralens are DNA intercalating molecules and their photo-excitation by UVA causes cross linking between DNA strands. This effect has been exploited since the ‘70 s, using 8-methoxypsoralen to treat psoriasis [182], and then the methodology has been extended to eliminate bacteria, viruses, like Chikungunya and dengue [183], protozoa in platelets and plasma blood components [184]. Interestingly, the inactivation with UVA seems to be augmented by the combination of psoralens with acidic lime extract [185]. Lastly, a new psoralen, amotosalen, UVA (320–400 nm) and INTERCEPT Blood System have been studied as protocol to photo-inactivate viruses, bacteria, protozoa and leucocytes in plasma for transfusions [186].

### Methylene blue

PSs belonging to this class are derivatives of a phenothiazinium ring, a significantly delocalized core which is particularly suitable to substitutions and alterations to change chemical and physical characteristics of the molecule [187–189]. For example, introducing changes that confer a different grade of lipophilicity to the molecules results in changes in dark toxicity and antibacterial effectiveness [190]. The red absorption is advantageous for deeper tissue penetration.

The best known dye is methylene blue which is especially relevant for its applications in plasma photo-disinfection, in particular, it is one of the compounds reported by WHO at the basis of the blood antiviral treatment [191], and as antimalarial agent [192]. Other components of the class are new methylene blue, dimethylmethylene blue and three dyes called azure A, B, and C [190]. The predominant area of use of methylene blue is as PS against fungi, whereas the related Toluidine blue has demonstrated a significant capability to photo-inactivate bacteria, as the multidrug-resistant *P. aeruginosa*, [193], also as biofilms [144, 194]. Methylene blue and Toluidine blue O appear to be able to carry out an antibacterial activity also in vivo, in murine methicillin-resistant *Staphylococcus aureus* arthritis [195] and peri- implantitis models [196], respectively. Although methylene blue is reported to produce ROS through both Type 1 and 2 mechanisms, photo-oxidation of nucleic acids proceeds essentially following the Type 2 process [197]. The use of methylene blue in viral photo-treatment has been documented [198], also on Zika virus [199] and enveloped viruses [200], including SARS-CoV-2 [201].

### Phenalenone derivatives

Phenalenones are synthesized by plants as a defense mechanism against pathogens using sunlight to generate ^1^O_2_ [202]. The phenalenone derivative 1H-Phenalen-1-one-2-sulfonic acid (PNS) was introduced and used as a reliable reference material. Positively charged and water-soluble phenalenone derivatives were introduced for antimicrobial applications [203].

### Xanthene derivatives

Xanthene is an organic compound that constitutes the core of various fluorescent dyes, such as fluorescein, rhodamine and eosin. Among them, some are PSs, such as Erythrosine, an iodinated derivative [204], that is approved as bacterial marker in odontology thanks to its ability to bind to biofilms [205] and that has demonstrated to be more effective than Photofrin and methylene blue in eradicating *Streptococcus mutans* biofilms [206]. The related compound Eosin is also an efficient PS [207].

Rose Bengal is a xantene iodinated derivative as well, but it has found application in ophtalmology. It is worth to remember that since ‘60 s Rose Bengal has been recognized to be effective on Gram-negative bacteria (*E. coli*) [208], both in solution and encapsulated in polystyrene beads [209]. Singlet oxygen production through a Type 2 mechanism allows Rose Bengal to induce damages on cellular membrane [210] and photo-inactivate both Gram-positive bacteria, like *Deinococcus radiodurans*, and Gram-negative bacteria, like *Aggregatibacter actinomycetemcomitans* [211].

An interesting application is the use of Rose Bengal in wash water decontamination, exploiting *Escherichia coli* BL21 and the bacteriophage T7 as model organisms [212].

### Crystal violet

A cationic triphenylmethane dye, called crystal or gentian violet, is a well-known local antiseptic [213], but by now superseded by more modern drugs. The name refers to its color, similar to the one of petals of gentian flowers. This molecule has a more relevant role as antifungal agent than antibacterial PS, because it is able to photo-kill methicillin-resistant *S. aureus*, but with low effectiveness [214].

### Cyanine derivatives

Indocyanine green is a PS belonging to the cyanines family. Exploiting its fluorescence and NIR absorption properties, indocyanine green was utilized as a marker in diagnostics, but it was demonstrated to be able to kill both Gram-positive bacteria, like wild type and resistant strains of *S. aureus,* and Gram-negative bacteria, like *P. aeruginosa* [215], *Porphyromonas gingivalis* and *Aggregatibacter actinomycetemcomitans* [216].

Unlike indocyanine green, that is a cationic derivative of cyanine, absorbing at 800 nm, Merocyanine 540 is neutral and shows a significant blue shift in absorption spectrum (555 nm). The efficiency as PS is not very high, both on planktonic cultures [217] and biofilms, requiring a significant light fluence and showing a limited radiation penetration [218].

### Dipyrrometheneboron difluoride (BODIPY)

BODIPY is the abbreviation of borondipyrromethene and indicates a class of fluorescent dyes whose structural core is constituted by 4,4-difluoro-4-bora-3a,4a-diazas-indacene. A number of derivatives have been developed to optimize absorption and fluorescence properties as well as photostability [219]. In particular, to improve their performance as PSs, functional groups [220] and heavy atoms [221] have been introduced. In spite of this, BODIPY derivatives show limitations in use as PSs, due to a scarce capacity of accumulation in malignant cells. However, two variants with one pyridinium cationic group on position 8 and two iodine atoms on 2,6-positions of the core proved to be effective in photo-killing Gram-positive *Staphylococcus xylosus* and the Gram-negative *E. coli* bacteria [222].

### Squaraine dyes

Squaraines are a class of organic dyes displaying an intense fluorescence emission in the red and far red region. They are derived from squaric acid through an electrophilic aromatic substitution reaction to form a highly conjugated system with extensive charge distribution. The structure is typical of a 1,3-zwitterionic donor–acceptor–donor system with an acceptor ring in the centre of the molecule and lateral donor aromatic rings, which, if substituted by heterocyclic or polyaromatic rings, modify the optical properties [223] and the stability of the molecule, making them usable in biomedical imaging. Moreover, bromo-, but mainly iodo- variants showed to be apt to use in antimicrobial PDI against Gram-negative *Salmonella typhimurium* [224].

### Fullerenes

Fullerene is a carbon allotrope where atoms arrange to form a closed or partially closed mesh which can assume different shapes, like empty sphere, ellipsoid or tube. One of the most famous components of this class is C60, that is constituted exclusively by 60 carbon atoms. Pure C60 shows features that represent advantages and disadvantages for application in antimicrobial PDT: it is stable, it sensitizes ROS through Type 1 and 2 mechanisms [225], C60 is non-toxic [226], but it is not soluble in aqueous solutions [227] and absorbs in UV and blue spectral region. To overcome the disadvantages, C60 derivatives have been synthesized with improved solubility [228] and singlet oxygen quantum yield. Another strategy to solubilize fullerenes, has been resorting to PEGylation, encapsulation in dendrimers, micelles, cyclodextrins and self-nano-emulsifying systems.

In particular, different cationic functionalized fullerenes have been developed and among these tri-cationic BF-6 [229] and the six quaternized cationic groups compounds [230] have proven to be strongly effective against *E. coli* and *S. aureus*. Moreover, DTC60 2 + , a dicationic fullerene composed of a hydrophobic carbon sphere, and two attached cationic groups that form a single monoadduct to the sphere [231] has demonstrated some selectivity and capability to photo-kill Gram-negative bacteria [232]. Interestingly, a new methodology has been fine-tuned allowing to obtain a set of fullerenes with five, 10 or even 20 cationic charges around a C60, or a C70 or a C84 cage [233] and some of them proved to be very promising antimicrobial PSs. The effectiveness of fullerenes against viral particles was proved in the dark [234] and upon irradiation [235], through both Type 1 and 2 mechanisms [236] and recently reviewed [29]. As previously observed for bacteria, also in antiviral PDI, constructs have been developed to overcome the low solubility limitations of fullerenes [237].

## Molecular targets of photodynamic inactivation

Antimicrobial PDI is characterized by a non-selective mode of action, where multiple targets are hit by the oxidant species. The molecular species interested by photo-oxidation comprise proteins, lipids and nucleic acids [238], but it is normally considered that photoinduced killing occurs mostly for damages at the level of cytoplasmic membranes and DNA [10, 239], The capability of a PS to induce these effects is in turn dependent on its localization, due to the short lifetime of photoinduced reactive species. To better appreciate how important the localization of the PS is in determining the outcome of its phototoxic action, it is important to put the above consideration in the context of the peculiar structures of bacteria and viruses.

### Bacterial structure

Gram-positive and Gram-negative bacteria have very different bacterial wall structures (Fig. 10). Gram-positive bacteria have a cell wall made of lipoteichoic and teichoic acids organized in multiple layers of peptidoglycan, with a thickness in the order of 30–100 nm. This outer layer of the wall is characterized by some porosity that enables penetration toward the interior of the cell of molecules like PSs and even macromolecules [240]. The cytoplasm is surrounded by a single lipid bilayer, mainly composed of phosphatidylglycerol (70–80%) and cardiolipin (~ 20%) [241, 242].

In the case of Gram-negative bacteria, the phospholipid inner bilayer is composed by 80% of zwitterionic phosphatidylethanolamine, ~ 15% of anionic phosphatidylglycerol and ~ 5% of anionic cardiolipin. The next layer is the 2–7 nm thick peptidoglycan layer, which is anchored to the outer membrane by lipoproteins. The outer leaflet of this membrane contains glycolipids, mainly negatively charged lipopolysaccharides, lipoproteins and β-barrel proteins [239, 242].

The outer membrane of Gram-negative bacteria imposes a tight barrier to molecular species coming from the extracellular matrix, and in general there are two ways to get through it, a lipid-mediated pathway for hydrophobic molecules, and general diffusion porins for hydrophilic molecules [243].

### Viral structure

**Fig. 11:**
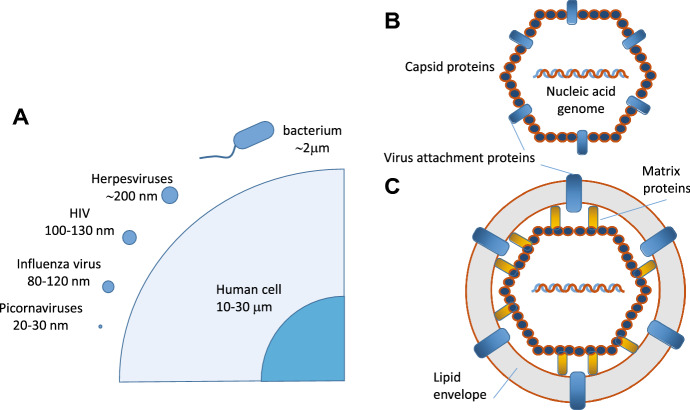
**a** Virus and cell size comparison (not to scale). Human viruses can vary in size but are generally in the range of 20–200 nm in diameter. In comparison, bacteria are generally 2–3 μm in length, and an average human cell is 10–30 μm. **b** Schematic of a naked virus structure. **c** Schematic of an enveloped virus structure [245]

Viruses are infectious agents that consist of a nucleic acid genome packaged within a protein shell. In spite of their relatively simple structure, viruses show a remarkable diversity in terms of size, genome organization, and capsid architecture [244]. To protect its genome from the environment, the virus surrounds its nucleic acid with a protein shell, called capsid, composed by one or a few proteins that self-assemble in a repetitive structure. Most viruses also have an envelope surrounding the capsid, a lipid membrane that is derived from one of the cell’s membranes, such as the plasma membrane (Fig. 11) [244, 245]. The majority of viral pathogens that cause emerging and re‑emerging infectious diseases are membrane-enveloped viruses, which require the fusion of viral and cell membranes for virus entry.

### Biological targets in bacteria and development of resistances toward photodynamic inactivation

The variety of molecular microbial targets, comprising proteins, lipids, and nucleic acids, makes it very unlikely that the microorganism can develop resistance toward the photodynamic action [10, 246, 247].

Development of resistance in microorganisms is more likely for methods that have one specific target structure following the key-lock principle like antibiotics or antiseptics [248, 249]. For such approaches, pathogens may overcome the pharmacological action by punctual mutations, expression of efflux pumps, or upregulation of defense-associated enzymes. On the contrary, since PDI is a non-selective approach with multiple targets in focus, it is much less likely that bacteria develop resistance [37].

Bacteria are often exposed to oxidative stress either of intracellular origin, or deriving from interactions with host cells and/or other pathogens. Molecular oxygen is rather unreactive due to its triplet state nature, but the electrostatic characteristics of this species allows it to readily cross biological membranes, including bacterial walls [250].

The four-electron reduction series of O_2_ implies the addition of consecutive electrons to generate superoxide (O_2_^−^), hydrogen peroxide (H_2_O_2_) and the hydroxyl radical (HO^•^). Accumulation of ROS, able to oxidize various biomolecules, creates a condition of oxidative stress. Bacteria have devised defense mechanisms based on enzymatic pathways like those catalyzed by superoxide dismutase, catalase, peroxidase and enzymes of the glutathione system [250].

These detoxification mechanisms may be able to provide bacteria with an adaptation against Type 1 photoreactions by upregulation of the enzymes expression [34, 247].

In view of the small size of the sphere of action for these ROS [52], oxidative damage only occurs at the site of the localization of a PS, which in many cases is restricted to the cell envelope. The long distance between the oxidative burst and the intracellularly located defense enzymes means that a significant action by the protective enzymes may be prevented [247]. In this direction, it was observed that superoxide dismutase activity was increased in *S. aureus* after treatment with protoporphyrin IX, but these strains were anyway photoinativated [251].

Different from ROS, ^1^O_2_ generated in Type 2 photoreactions has no protection mechanism by specific enzymes. Furthermore, it is very improbable that bacteria could develop such a system as ^1^O_2_ is not an oxygen radical, but just molecular oxygen in a higher energetic state (0.98 eV above the ground state). Nevertheless, it is worth mentioning that carotenoid quenchers are synthesized upon exposure of photosynthetic systems to exceedingly high light intensity [252].

Bacteria have an additional defense system, consisting in efflux pumps that extrude potentially toxic compounds to the extracellular space [253], that may become relevant also for developing resistance toward antimicrobial PDI. Efflux pumps were indeed found to reduce the intracellular concentration of methylene blue and its associated ROS concentration [254, 255]. No other PSs have been reported to date as potential substrates of efflux pumps.

Possible induction of resistance to a specific treatment is usually studied by applying a sub-lethal dose of the therapy and by subsequent culture of bacteria that have survived [239]. Only a few attempts to expose resistance to PDI in bacteria have been reported in the literature [256–258]. In PDI studies on *Aggregatibacter actinomycetemcomitans* and *Peptostreptococcus micros*, bacteria were not found to develop resistance after 10 repeated cycles. [256] Resistance was not induced on studies on *E. coli* and antibiotic-sensitive and resistant strains of *S. aureus* (MSSA and MSRA), tested through 11 (*E. coli*) or even 25 passages (MSSA, MRSA). [259] Similarly, no resistance was induced in strains of *Vibrio fischeri* and *E. coli* after 10 cycles of photodynamic treatment [258] nor was after 20 photoinactivation cycles on *S. aureus*, *Pseudomonas aeruginosa* and *Candida albicans* [257].

### Viral targets of photodynamic inactivation

It is possible to identify three main molecular targets in the general structure of viruses for the reactive species photosensitized in photodynamic treatments. They comprise nucleic acids (DNA or RNA, depending on the viral type), virus proteins and, for enveloped viruses, viral lipids (Fig. 11) [260] [32]. Photoinduced damages on viral nucleic acids and proteins have been thoroughly investigated [32, 261, 262].

Since the lipid bilayer offers an additional target for ROS, enveloped viruses appear to be more sensitive to PDI than those without an envelope. [235, 263–265] While a detailed analysis of the effects of PDI on viral lipids is lacking, several evidences have been accumulated in photodynamic treatments of tumors [266]. Inhibition of the fusion step has been described by lipid membrane targeting [267, 268]. The photoinduced damages to the phospholipids in the viral membrane affect both the curvature and fluidity of the membrane. Upon generation of ^1^O_2_ in close proximity of the viral membrane the C=C double bonds of unsaturated phospholipids are oxidized with *cis*-to-*trans* isomerization and introduction of hydroperoxy (–OOH) groups. This leads to increased positive curvature and reduced fluidity of the membrane, which in turn influence negatively the capability of viral membranes to undergo fusion with the plasma membrane [267–269].

The phospholipid components of biological membranes are characterized by a cylindrical shape, endowed with a polar head and a hydrophobic tail. They self-assemble to form bilayers which are planar in nature, but may undergo bending, e.g. to form liposomes, at an energetic cost. Using the physicochemical properties of a lipid bilayer [270] it is possible to estimate the energetics for this process [268].

**Fig. 12:**
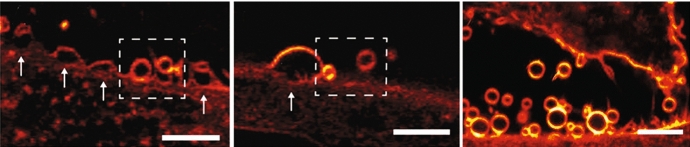
Close-up images on swelling and vesicle formation as a consequence of the photoinduced damage on the plasma membrane on HeLa cells loaded with Hyp 0.5 μM during STED image collection (left and center). The plasma membrane swells toward the extracellular medium at those points marked by a white arrow. Spherical vesicles are detaching from the plasma membrane in the area surrounded by the dashed line (right). Scale bars, 10 μm. Figure adapted with permission from: P. Bianchini, M. Cozzolino, M. Oneto, L. Pesce, F. Pennacchietti, M. Tognolini, C. Giorgio, S. Nonell, L. Cavanna, P. Delcanale, S. Abbruzzetti, A. Diaspro, and C. Viappiani, Biomacromolecules, 20, 2024–2033 (2019). Copyright 2019 American Chemical Society

The energy per unit surface which is necessary to create a sphere starting from a flat surface can be written as:

6 $$G_{{\text{b}}} = \frac{2\kappa }{{r^{2} }},$$

where *κ* is the membrane rigidity and r is the radius of the sphere. The rigidity *κ* can be expressed in terms of *k*_B_*T*, where *k*_B_ is the Boltzmann constant and *T* is the absolute temperature. The energy cost for bending a membrane increases dramatically when formation of spheres of small radius (i.e. high curvature) is involved, such as those that occur during formation of the fusion stalk during viral infection. Modeling of this process suggests that minor increase in membrane rigidity, such as those caused by lipid oxidation produced in photosensitized production of ^1^O_2_ by lipophilic PSs, may impair the process [267, 269]. Damages to lipid bilayers are difficult to visualize in bacteria and viruses, but are more readily visualized on plasma membranes, where photo-oxidation induced by lipophilic PS leads to extensive damage that results in blebbing and membrane permeabilization. This is evident in Fig. 12 where close-up STED images on the plasma membrane portions of HeLa cells loaded with Hyp are shown [271].

The in vitro methods used for testing antivirals differ largely in the literature depending on the viral target and have been recently reviewed [272]. In vivo models for assessing the antiviral efficacy have been also described [18, 273].

An added value of antiviral PDI stems from its untargeted mechanism of action which is robust against development of resistance, an issue that has been reported about antiviral drug given the genetic flexibility of viruses, and is a current cause of concern [274, 275].

As in the case of bacteria, PDI of viruses has been mainly applied to localized viral lesions [11, 13, 276, 277].

It is worth mentioning that activation of a systemic response of the immune system has been reported in cancer applications [278, 279], a fact that opens new perspectives also for antiviral PDI.

## Delivery of photosensitizers and theranostic formulations

Several existing photosensitizing compounds suffer from low water solubility which results in aggregation of the compounds into photochemically inactive structures and reduces their bioavailability. The use of water soluble carriers allows to bypass this issue, by providing suitable local environments where aggregation is prevented and the photophysics of the compounds is preserved [97, 280–283]. The delivery strategies cover a wide array of concepts ranging from stimulus-responsive switchable molecular photosensitisers to proteins as novel photoactive biotherapeutic drugs to metallic nanostructures as plasmonic antennas for ^1^O_2_ [97].

**Fig. 13:**
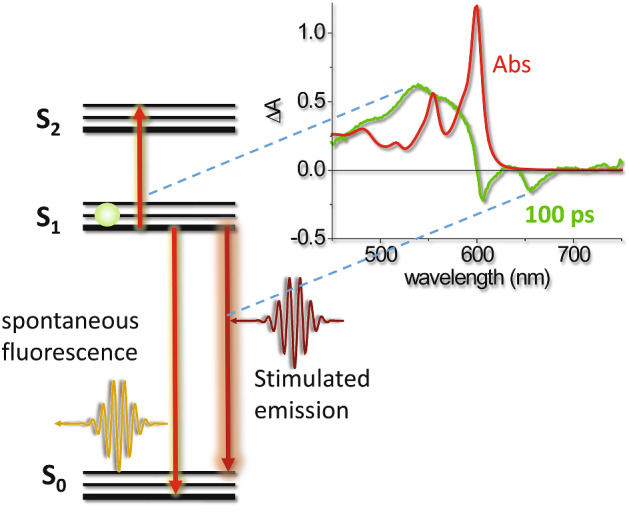
Stimulated emission by Hyp is observed in femtosecond pump-probe experiments [284]. The red curve reports the absorbance spectrum of Hyp in DMSO, the green curve is the differential absorption spectrum collected at 100 ps after excitation and corrected for ground state bleaching. excited state absorption is observed at ~ 550 nm, stimulated emission is observed at ~ 600 and ~ 650 nm. The absence of excited state absorption in the far red-near infrared region warrants the possibility of inducing stimulated emission through excitation in this spectral range without the concomitant excitation to higher energy electronic states. This principle is at the basis of the application of Hyp in STED nanoscopy (Fig. 14). Femtosecond pump-probe data reproduced from Delcanale et al. [284]

**Fig. 14:**
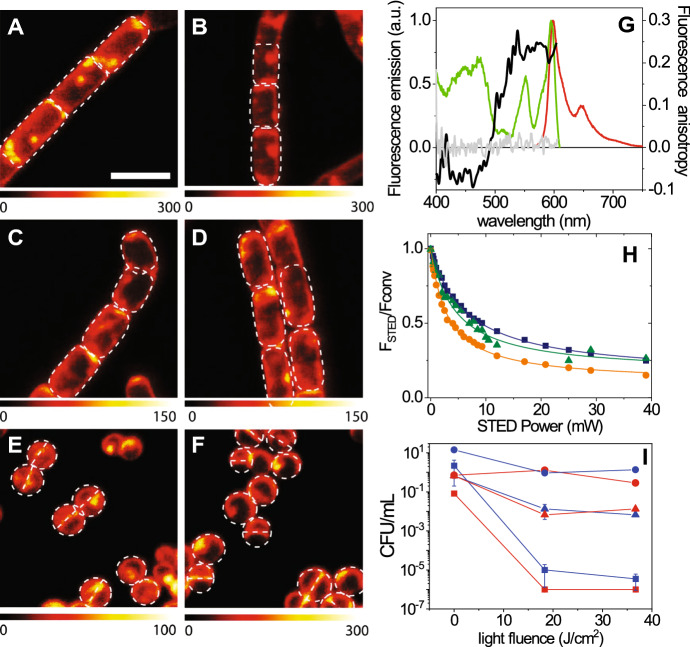
Distribution of Hyp in different types of bacteria as revealed by STED nanoscopy [284]. Selected STED images of *B. subtilis* (**a**, **b**), *E. coli* (**c**, **d**) and *S. aureus* cells (**e**, **f**). Hyp was delivered as a complex with apoMb. Images were collected under excitation at 566 nm and detection at 605/70 nm. The STED beam was at 715 nm, power 30 mW and dwell time 0.1 ms (**a**–**d**) and 0.05 ms (**e**, **f**). White dashed lines are intended as visual aid to guide the eye along the bacterial shape. Scale bar, 2 μm. **g** Fluorescence excitation (green) and fluorescence emission (red) spectra for the complex Hyp-apomyoglobin in PBS buffer. The grey line is the fluorescence anisotropy excitation for a DMSO solution of Hyp, the black line is the fluorescence anisotropy excitation for the complex Hyp-apomyoglobin in PBS buffer. H Fluorescence depletion curves for Hyp in DMSO (orange), Hyp-apomyoglobin in PBS (blue squares), and Hyp bound to liposomes (green triangles) collected under excitation at 570 nm and detection at 605\70 nm. The STED beam was at 715 nm. Solid lines are the best fit to the depletion functions [285]. I. *S. aureus* (squares), *B. subtilis* (triangles), and *E. coli* (circles) photoinactivation after incubation with Hyp (red) or Hyp-apomyoglobin (blue). Figure adapted from P. Delcanale, et al., Scientific Reports, 5, 15564 (2015) [284]

Delivery of photosensitizing compounds with water soluble carriers ideally preserves the quantum yield of triplet state formation, necessary for in situ photo-generation of ROS. In addition, solubilized PSs often show fluorescence emission, which conveniently enables a direct detection of these species with microscopes or in vivo imaging systems. Due to the competition between the physical processes of intersystem crossing, leading to formation of molecular triplet state, and radiative decay, leading to emission of fluorescence, the observed fluorescence quantum yields of PSs are usually moderate or low. The case of Hyp is a relevant example showing the advantage of using carriers able to preserve molecular photophysics. When this PS is solubilized in combination with a carrier, the fluorescence and intersystem crossing quantum yields are largely preserved. Moreover, the molecular excited singlet state can be efficiently depleted through stimulated emission by means of a far red laser pulse with high power density (Fig. 13). As a consequence, the spontaneous fluorescence emission occurring by radiative decay from the excited singlet state is also depleted, as shown in Fig. 14 (panel h).

Thanks to this effective excited state depletion, Hyp delivered with protein-based carriers was localized on bacterial cells with stimulated emission depletion (STED) super-resolution microscopy (Fig. 13) [286], achieving a lateral spatial resolution of ∼ 90 nm, well-below the diffraction limit expressed by the Abbe’s law (∼ 250 nm for visible light) [284]. In addition, Hyp retained an effective photoinduced generation of ROS and a significant antimicrobial activity against Gram-positive bacteria. The possibility to reach such subdiffraction resolutions is particularly important for antimicrobial applications, due to the relatively small size of bacterial cells (1 μm), which often prevents a clear localization of photoactive agents at subcellular level.

Using this approach, the delivery of photosensitizing drugs to bacterial cells is directly visualized, without introduction of exogenous labels, and dynamic information can be extracted, by monitoring changes in the localization and intensity of the photosensitizing compound during the treatment [287]. Moreover, simultaneous detection of both the photosensitizing compound and the carrier during the delivery process is readily achieved, for example by labeling the carrier with a spectrally distinct fluorophore [68]. Despite fluorescence emission is conveniently used for imaging, diverse theranostic constructs can be obtained by combining PSs with species that give other types of diagnostic readouts [288, 289]. In spite of their potential, applications of fluorescence imaging techniques with subdiffraction resolution to investigate the interaction of the PS with their targets and the effect of photosensitization on the microbial structure are still just a few.

### Passive carriers

In view of their high biocompatibility, the use of small size, water soluble proteins where hydrophobic docking sites or clefts exist, is a powerful strategy to overcome low water solubility of photosensitizing molecules, enhancing bioavailability of carried PSs [290]. Exploiting proteins as biocompatible carriers appears even more appealing when combined with naturally occurring PS molecules, whose use against bacteria has been reviewed [291]. Easily obtained water soluble proteins such as apomyoglobin (i.e. myoglobin where the prosthetic group heme has been removed, apoMb) [271, 284, 292, 293], β-lactoglobulin (βLG) [294, 295], and serum albumins (e.g. human, HSA, or bovine, BSA) [68, 296] have been exploited to transport several hydrophobic PS, including naturally occurring PSs like Hyp and curcumin.

Interaction between photosensitizing molecules and protein cavities or clefts of apoMb, HSA, BSA, or βLG is characterized by weak forces and results in dissociation constants in the order of 1–10 μM for Hyp, [68, 293–295] or even higher (in the order of 100 μM) for curcumin [296].

**Fig. 15:**
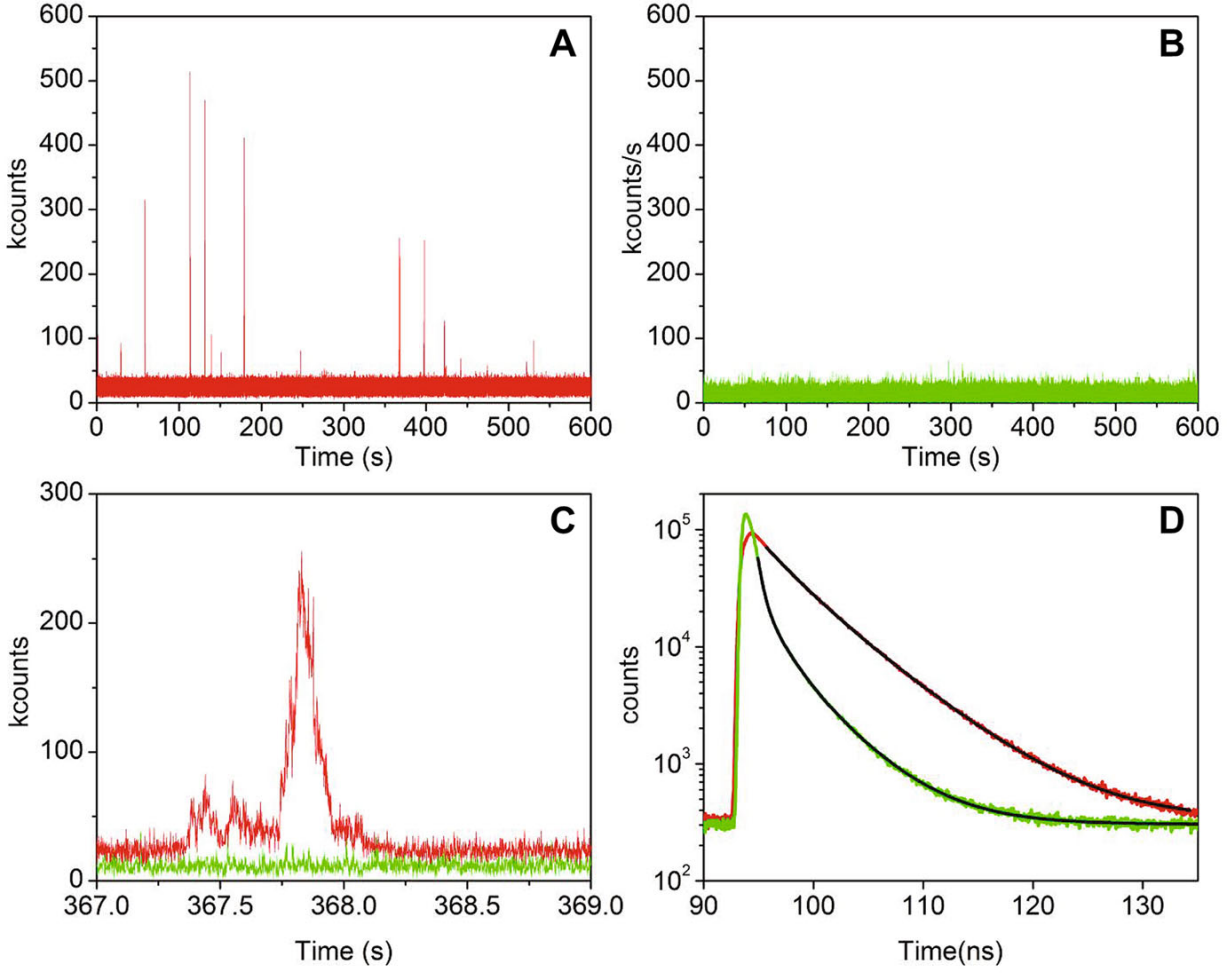

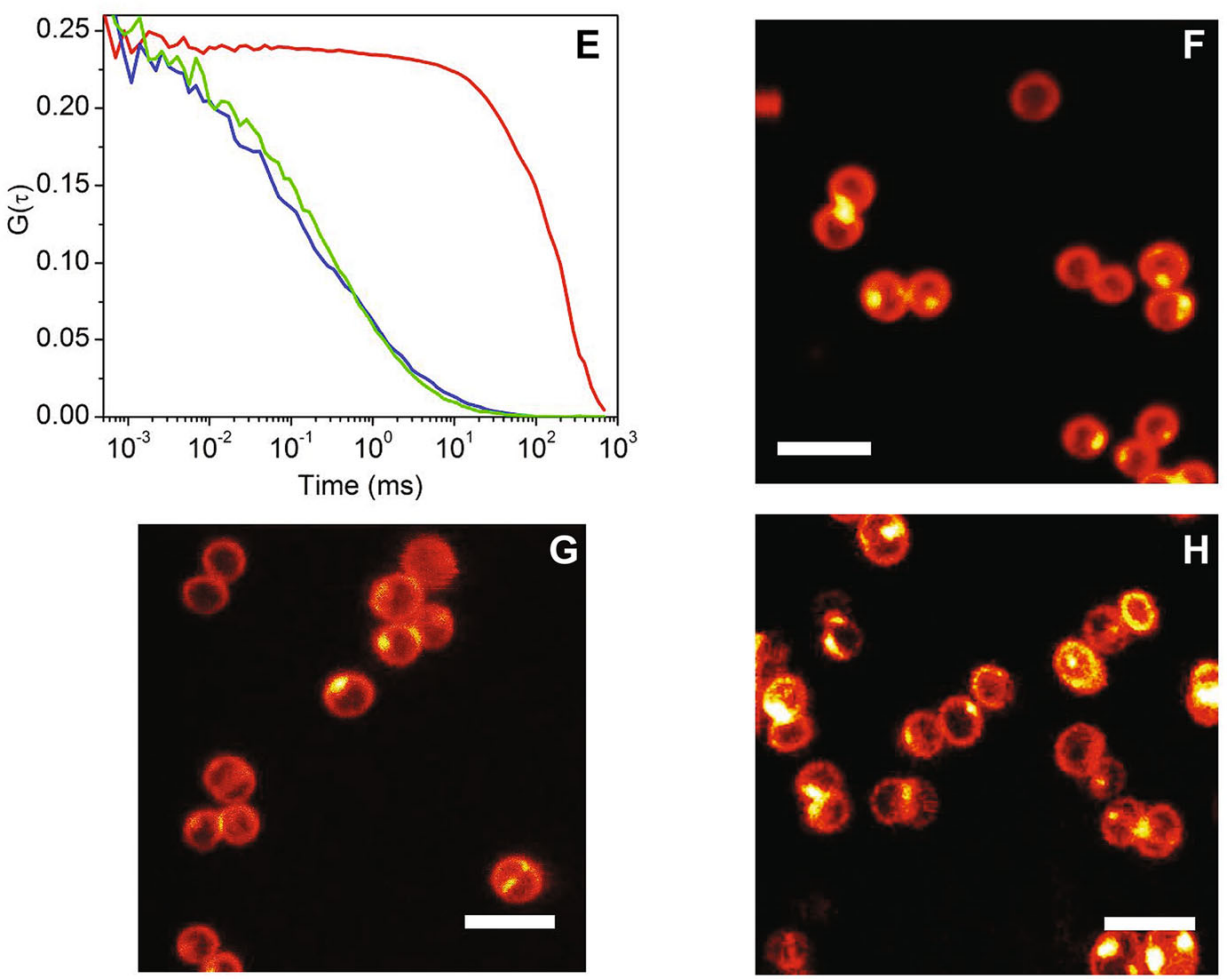
Delivery of Hyp to *S. aureus* by means of serum albumin labeled with fluorescein isothiocyanate (FITC). [68] Time traces collected with an FCS setup for fluorescence emission in the red (**a**) and in the green (**b**) for a 600 s acquisition on a *S. aureus* suspension loaded with Hyp bound to FITC-BSA. Time bins are 1 ms wide. (Hyp 100 nM, BSA 30 μM, FITC-BSA 100 nM). **c** Expanded view on selected portions of the MCS traces in **a**, **b**. **d** TCSPC histograms calculated for the full acquisition time in **a** (red curve) and **b** (green curve). The black curves are tail fits to a double exponential decay (for the red curve) with lifetimes *τ*1 = 2.96 ns (40%) and *τ*2 = 6.10 ns (60%) or a triple exponential decay (for the green curve) with lifetimes *τ*1 = 0.48 ns (60%), *τ*2 = 1.88 ns (29%) and *τ*3 = 4.60 ns (19%). **e** Cross-correlation function (red curve) calculated for the trace in panel **a**. Best fit is obtained with a diffusional model plus a triplet state decay. The diffusing species is characterized by a diffusion coefficient *D* = 0.3 μm^2^/s (consistent with diffusing objects the size of *S. aureus*) and the triplet decay by a lifetime of about 20 μs. The green curve is the cross-correlation curve obtained for the trace in panel **b**. Best fit is obtained with a diffusional model plus a triplet state decay. The diffusing species is characterized by a diffusion coefficient *D* = 60 μm^2^/s, consistent with the expected value for BSA, and the triplet decay has a lifetime of about 20 μs. The blue curve is the cross-correlation curve obtained for Hyp-BSA in PBS buffer (Hyp 100 nM, BSA 30 μM) in the absence of bacteria, monitoring emission in the red. The diffusing species is characterized by a diffusion coefficient *D* = 60 μm^2^/s, indicating that Hyp is bound to BSA. F. Selected STED image of *S. aureus* cells in the presence of Hyp (1 μM) collected under excitation at 566 nm and detection at 605\70 nm. The STED beam was at 715 nm, power 30 mW and dwell time 0.05 ms. Scale bar, 2 μm. **g** Selected STED image of *S. aureus* cells in the presence of Hyp-apoMb ([Hyp] = 1 μM, apoMb = 3 μM). Conditions as in **f**. **h** Selected STED image of **b**. *S. aureus* cells in the presence of Hyp (500 nM) bound to BSA (5 μM) collected under excitation at 560 nm and detection at 570–670 nm. The STED beam was at 775 nm, power 130 mW and scan speed 8000 Hz. Scale bar, 1 μm, 128 averages. Gating windows from 1 to 7 ns. Figure reproduced with permission from Pezzuoli et al. [68], copyright Elsevier Ltd

This property may be detrimental to bioavailability in systemic administration, since Hyp can translocate to other abundant proteins like, e.g., serum proteins, or the lipid phases of cellular membranes in real biological systems. On the other hand, in topical applications, this property may be beneficial for a fast delivery to the microbial structures and allow reduced Drug-to-Light Interval (DLI). A short DLI in topical applications may favor antimicrobial PDI by reducing side damages to the host tissue, where the uptake of the drug may be generally slower. Transfer from the protein carrier to the bacterial wall was demonstrated using FCS and a two-color detection system [68]. Hyp was bound to FITC-labeled BSA, and the resulting autocorrelation curves for the green emission (from the BSA bound FITC) and the red emission (from Hyp) were collected. The same diffusion coefficient (*D* ~ 60 μm^2^/s) was extracted from the red and the green autocorrelation curves, indicating both fluorophores are associated with BSA. In the presence of bacteria, the diffusion coefficient obtained from the green emission was unchanged, whereas analysis of the autocorrelation function for the red emission afforded a diffusion coefficient *D* ~ 0.3 μm^2^/s consistent with that expected for an object of the size of bacteria, demonstrating transfer of Hyp from the carrier protein to *S. aureus* (Fig. 15) [68].

Other types of passive carriers not based on a protein scaffold, consist in formulations where PSs are associated to an artificial nanostructure, such as liposomes [297] or nanoparticles [298]. Unlike protein-based carriers, these systems are loaded with multiple copies of photosensitizing molecules that are tightly bound to the carrier (by chemical conjugation, encapsulation or adsorption) and less likely to be spontaneously released. The antimicrobial efficacy is primarily related to the passive accumulation of the nano-carriers on bacterial cells or to an enhanced photosensitizing activity in response to mutated environmental conditions, such as pH or temperature, achieved through specific designs [299].

### Carriers with targeting properties

The negatively charged bacterial wall (composed by phospholipidic bilayer and peptidoglycans, respectively, rich in phosphate and hydroxyl groups that carry a high density of negative charge at physiological pH) has attracted the attention of scientists working in the development of antibacterial photosensitizing agents, as this provides a strong electrostatic driving force toward positively charged molecular species that can in principle be exploited to achieve a certain degree of targeting. For example, several PSs functionalised with small cationic functional groups have been reported [300–303].

Cationic PSs such as methylene blue [304] and other phenothiazinium derivatives [305], or cationic porphyrins [306] and phthalocyanines [307] have been successfully employed and demonstrated higher efficiency, also with Gram-negative bacteria, than neutral or negatively charged molecules, that may require chemical modification to eliminate electrostatic repulsion.

Nevertheless, the selectivity of the above principle based on electrostatic interactions is limited and does not fully protect against phototoxic side effects on mammalian cells in tissues where the infection is localized. Recent strategies in antimicrobial targeted delivery have been reviewed [308].

**Fig. 16:**
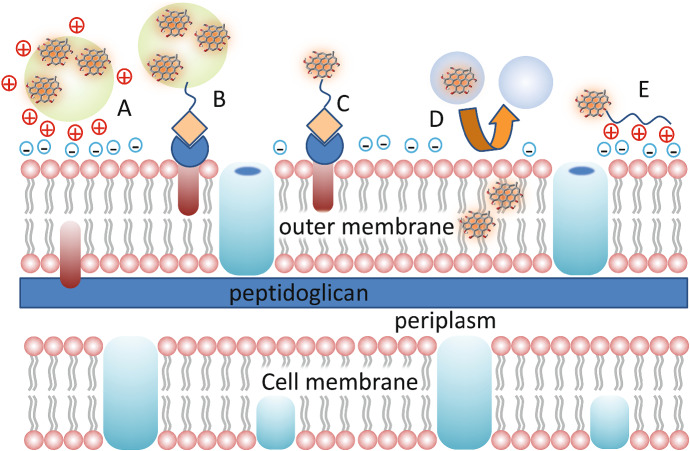
Cartoon summarizing some of the strategies for delivery of photosensitizers to bacteria (in this case a Gram-negative bacterium) [308]. **a** Positively charged nanostructures targeting the negatively charged membrane. **b** Antibiotic conjugated nanostructure. **c** Antibiotic conjugated photosensitizer. **d** Passive delivery by water soluble proteins. **e** Photosensitizer conjugated to positively charged peptide

**Fig. 17:**
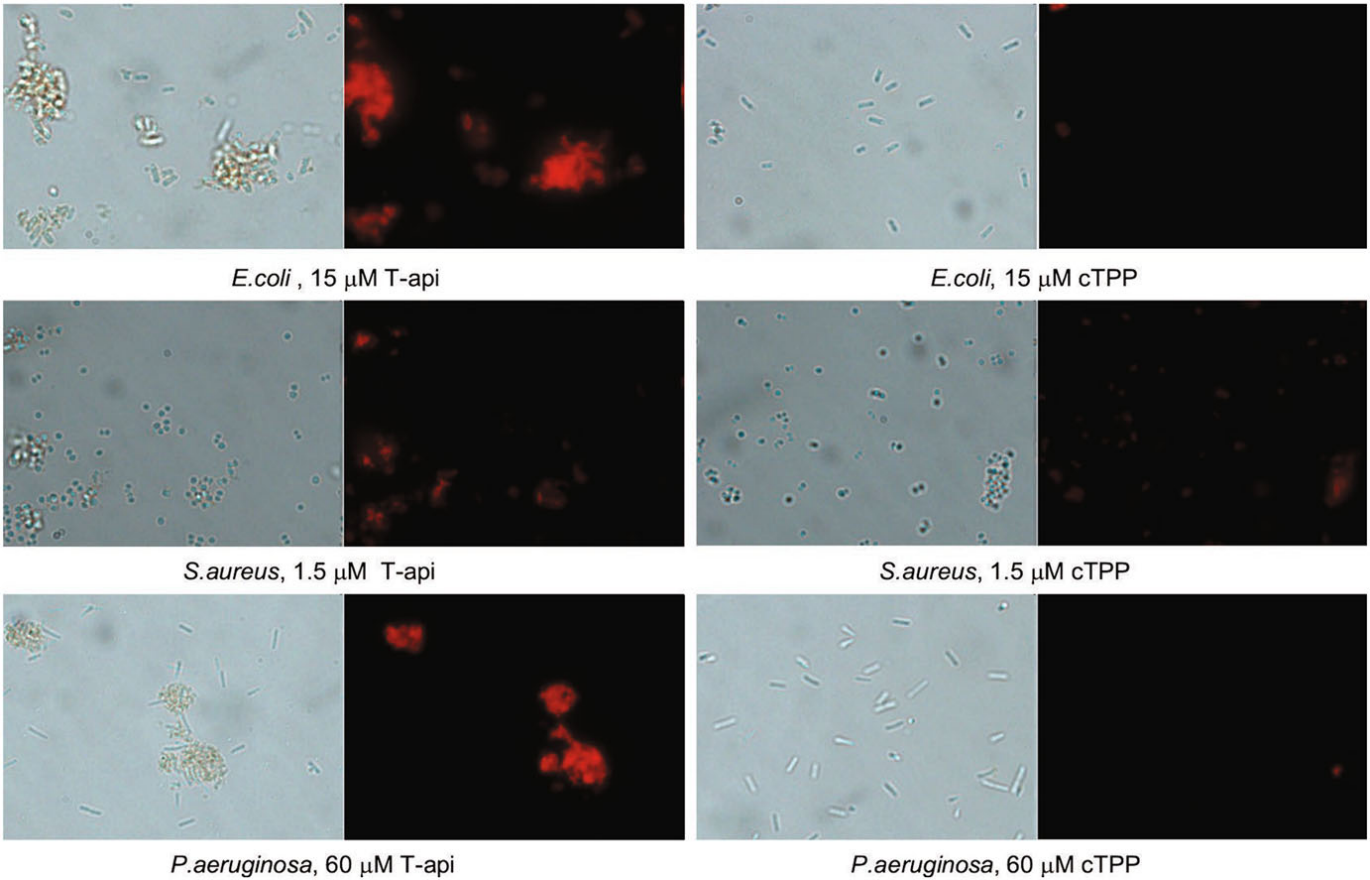
Fluorescence and corresponding bright field images of *E. coli*, *S. aureus* and *P. aeruginosa* incubated for 60 min with the indicated concentrations of T-api (left panels) or cTPP (right panels). Reproduced with permission from Dosselli et al., Copyright (2010) American Chemical Society [313]

Improvement in bioavailability and selectivity has been searched using several different strategies, some of which are summarized in Fig. 16. The delivery units comprise poly-cationic materials, bacterial-targeting peptides [309–313], polymers [314], antibiotics [315], antibodies [316–320] or other proteins [321, 322], such that the supramolecular structure acquires higher affinity for specific bacterial components (proteins, sugars,…) thus achieving passive or active targeting [313, 319, 323, 324]. Figure 17 shows an example of targeting achieved by a small cationic antimicrobial peptide (apidaecin Ib) conjugated to the PS 5(40-carboxyphenyl)-10,15,20-triphenylporphyrin. Fluorescence from the PS co-localizes with the bacteria only when it is bound to apidaecin Ib, a fact that results in efficient photoinactivation against tested bacterial strains [313].

Other strategies for targeted delivery to bacterial cells involve the use of nanostructured carriers. In this case, the nanomaterials act as scaffolds that accommodate multiple photosensitizing molecules while targeting of bacteria is conferred with surface modifications with functional bacteria-targeting units [298, 308].

The design of delivery systems with bacteria-targeting properties is very relevant to achieve effective theranostic applications, for which it is imperative to minimize off-target effects.

**Fig. 18:**
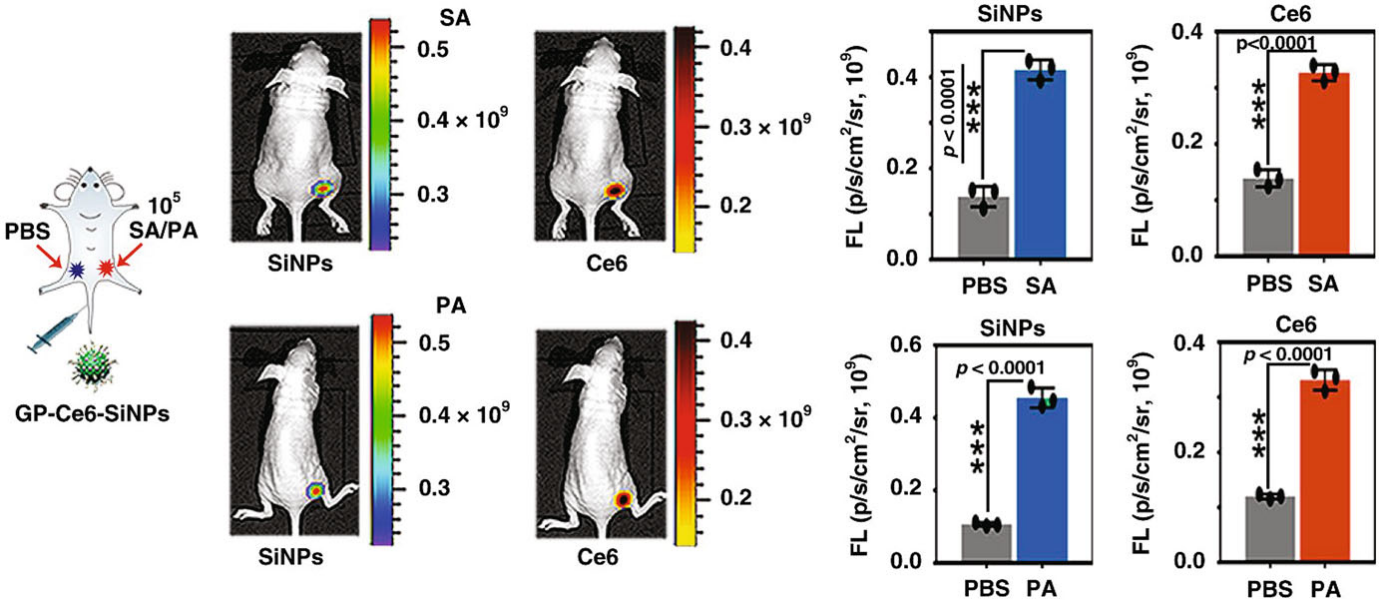
In vivo dual-emission imaging of 1.0 × 10^5^ CFU of *S. aureus* (SA) or *P. aeruginosa* (PA) and PBS-treated sites of mice (right and left sides respectively) injected with silicon nanoparticles bearing chlorin e6 and decorated with glucose polymers (GP-Ce6-SiNPs). The corresponding histograms of fluorescence intensity from silicon nanoparticles (SiNPs) and chlorine e6 (Ce6) at two sites of the mice are reported. Figure adapted from J. Tang, et al., Nature Communications, 10, 4057 (2019) [325]

In this context, a targeted antimicrobial theranostic agent was realized using small (< 10 nm) silicon nanoparticles decorated with glucose polymers and bearing adsorbed chlorin e6 as PS. The intrinsic green fluorescence emission of the nanoparticles and the far-red emission of chlorin e6 were exploited to localize the nanoagent in vitro, on bacterial cells, and in vivo, upon intra-venous administration in mice infected with *S. aureus* and *P. auruginosa* (Fig. 18). The small size of the construct, together with the surface modifications enable targeting and active internalization of the nanoagent by both Gram-positive and Gram-negative bacteria, through the ATP-binding cassette transporter, with a high specificity over mammalian cells. Significant photoantimicrobial effects were observed at high concentrations (10 mg/mL) and upon relatively intense photo-excitation of chlorine e6 (660 nm; 12 mW/cm^2^ for 40 min) [325].

## Selectivity of phototoxic action

As already discussed, the selectivity of PDI derives from the application of the illumination beam only to the interested tissues, where the short range action of the photoinduced ROS restricts the photooxidative effects to the close vicinity of the photosensitizing molecules. In addition, interaction of PSs with bacterial wall and/or internalization occurs on a relatively short time scale (small DLI) [326], so that illumination can be applied before the PS is uptaken by the host tissue, thus reducing unwanted damage [13]. Photoantimicrobials for local or topical treatments are thus endowed with selective therapeutic advantages over conventional drugs.

**Fig. 19:**
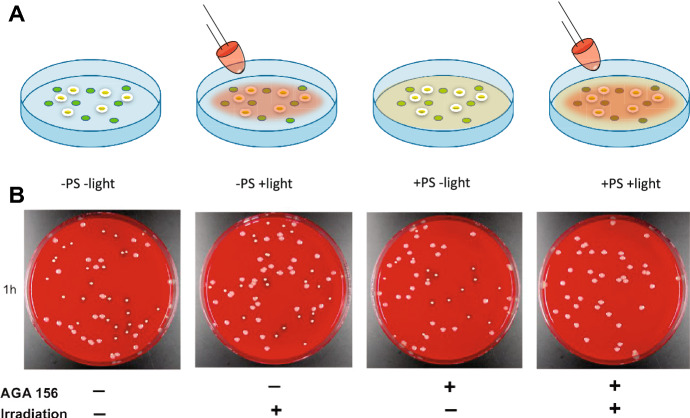
**a** Viable bacteria of different strains (Gram-positive, green dots; Gram-negative white dots) on a Petri dish (−PS −light) can be exposed to visible light (−PS −light) with no sizeable effects on their viability. Upon incubation with a suitable PS (+PS −light) no effects on viability are observed in any of the two strains, and only when visible light of suitable wavelength is shone in the presence of PS a decrease in viability of the Gram-positive strain (brown dots) is observed (+PS +light). Gram-negative bacteria are unaffected by the treatment. **b** Experimental demonstration of the above principle with *S. aureus* and *E. coli* in the presence of Si-phthalocyanine derivatives. Panel **b** reproduced with permission from Galstyan et al. [136]

When combined with targeting of bacterial components, selectivity is further enhanced. As an example, the higher efficiency of some PS against Gram-positive bacteria, has been exploited to selectively kill them when in the presence of Gram-negative bacteria. This was demonstrated for different photoantimicrobials functionalized with triphenylphosphonium [303], and for carbohydrate-conjugated silicon(IV) phthalocyanines [136]. The latter case is demonstrated in Fig. 19. The different efficiency may be of use where selective photoinactivation of Gram-positive pathogens is necessary, but preservation of commensal microflora is desired.

## Applications to biofilm eradication

In most disease conditions associated with microbial infection, bacterial cells are not free floating in biological liquids (planktonic) but rather organized into adherent structures with high cell density, that are called biofilms. These consist in a community of bacteria, belonging to a single or multiple species, that organizes in cell layers embedded in a viscous extra-cellular matrix, negatively charged, formed by extracellular polymeric substances [327, 328].

Eradication of bacteria in biofilm is considerably more challenging than eliminating planktonic cells. First, the biofilm environment provides an additional barrier to the diffusion of drugs, which may fail to reach the inner layers and be more prone to remain trapped in the matrix and degraded. Additionally, bacteria in the interior of biofilms are exposed to less nutrients and reduced oxygen concentration, if compared to planktonic cells, and are likely to develop more resistant phenotypes in response to such stress conditions, by upregulation of stress-response genes or overexpression of shock proteins and efflux pumps. Also, a population of *persister* cells can develop in the deepest layers of mature biofilms. These cells are in a dormancy state, non-growing and with reduced metabolic activity, which make them inherently able to escape antibiotics that target metabolic and cell division processes, but can grow rapidly when unfavorable conditions are removed [329, 330].

For these reasons, photodynamic treatment of biofilms is a promising alternative to antibiotics but often requires higher concentrations of photoactive agent and additional efforts to destabilize the matrix structure and/or enhance the penetration of the PS inside the biofilm [330].

A way to improve anti-biofilm activity of existing photosensitizing molecules, especially against Gram-negative bacteria, consists in the pre-treatment or co-administration of PSs with adjuvant agents that affect the structure of the bacterial cell wall or the biofilm matrix. An example includes the co-administration of methylene blue with a mixture of water, glycerol and ethanol for the disinfection of the root canal system of tooth specimens presenting *E. faecalis* biofilms. In this case, these adjuvants also promoted the solubilization of the PS and its local diffusion in situ [331]. Similar strategies use permeabilizing agents that destabilize the cell wall structure of Gram-negative bacteria, such as the polycationic polypeptide polymyxin B [332, 333] or EDTA.

In absence of adjuvant agents, the photodynamic treatment on biofilm should rely on a photosensitizing compound that is intrinsically able to penetrate the biofilm, preserving its activity.

Mainly in view of the increased viscosity and density of the biofilm matrix, together with its overall negatively charged environment, it is generally believed that small PSs (having molecular weight roughly below 1000 Da) that bear cationic charges are more likely to diffuse inside the biofilm structure and eradicate bacteria upon photo-activation. However, it should be noted that the final outcomes of photosensitization-based treatments on biofilms depend on a wide set of variables (type of bacteria, biofilm growth conditions, PS concentration, yield of ROS generation, spectral overlap with light sources, incubation time, media composition and light exposure) which make a direct comparison of the performances of different photosensitizing molecules particularly delicate. Therefore, even if several reports highlight the efficacy of small cationic molecules in anti-biofilm applications, it remains simplistic to draw general conclusions.

Small cationic phenothiazinium PSs, like methylene blue, toluidine blue O, azure A or rose bengal showed good photo-activity against both Gram-positive and Gram-negative bacteria in biofilm [334]. Among these photosensitizing molecules, methylene blue (284 Da, + 1 charge), combined with red light activation, is certainly one of the most widely employed [198, 335, 336], also for clinical applications [337–339]*.*

Porphyrin-based PSs are another popular class of compounds tested for biofilm eradication. These molecules are bigger (MW 500–2000 Da) than phenothiazinium PSs and a variety of compounds have been realized, with chemical modifications that modulate the molecular electrostatic charge. In general, several porphyrin-based compounds with positive charges were effective against Gram-positive and Gram-negative bacteria in biofilms [301, 340–342]. However, porphyrins with negative charges such as chlorin e6 [343] or photodithazine [344] also showed good results against biofilms of Gram-positive bacteria at high concentrations (200 μM and 1 mM, respectively).

Even hydrophobic PS compounds like the naturally occurring curcumin was reported to have anti-biofilm activity toward Gram-positive bacteria in vitro [345, 346] and in animal models [347, 348].

As previously described, the ability of a PS to effectively eradicate biofilms can be enhanced by the combination with carrier moieties. For example, a nanoparticle formulation of Hyp (45 nm diameter) was able to completely eradicate infection in rats presenting wounds infected with methicillin-resistant *S. aureus* [349]. This result is interesting considering that, in absence of carriers, Hyp showed only moderate anti-biofilm activity [350], even though results were obtained under different conditions and for different types of bacteria.

All in all, the results collected in this section show that photosensitization has a good potential for treatment of bacterial biofilm, alternative to antibiotics. Many different photosensitizing compounds with a diversity of modifications and formulations have been tested and several showed a promising antimicrobial activity against biofilms. In perspective, quantitative investigations that better address the mechanism of action of these compounds (diffusion, molecular interactions, uptake, …), enabling a more rational development of these treatments, are needed.

## Optical absorption and light sources

**Fig. 20:**
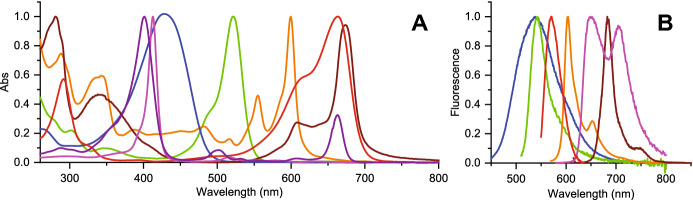
Normalized absorption (**a**) and emission (**b**) spectra of some representative photosensitizers. Curcumin in ethanol (blue); eosin in PBS buffer (green); TSPP in PBS buffer (magenta); Hyp in DMSO (orange); methylene blue (red), zinc phthalocyanine in DMSO (dark red)

Photosensitizers currently in use show absorption bands covering the whole visible spectrum and the near infrared. Most of them have appreciable absorption bands extending to the near ultraviolet. Figure 20a shows examples of absorption spectra of photosensitizing molecules, normalized to the most intense band. Normalized fluorescence emission spectra are reported in Fig. 20b.

Absorption is a fundamental property of the compounds as it starts the overall process of photosensitization [48]. The probability of the absorption process is determined by the optical properties of the molecule, through the molar extinction coefficient (with values typically in the range 10^4^–10^5^ M^−1^cm^−1^), which is in turn related to the dipole transition moment for the considered electronic transition. An important aspect in determining how effective the illumination is in photosensitizing ^1^O_2_, is the number of absorbed photons by the PS. Regardless of the photon energy, the number of absorbed photons, through intersystem crossing and ^1^O_2_ quantum yields, represents how many precursors of the ROS are generated and provides a more correct estimate of the light dose [351]. As sketched in the Jablonski diagram (Fig. 2), excitation in any of the absorption bands results in subsequent energy loss, bringing the molecule to the lowest electronically excited state (*S*_1_) and then eventually the triplet state *T*_1_ [48–50].

**Fig. 21:**
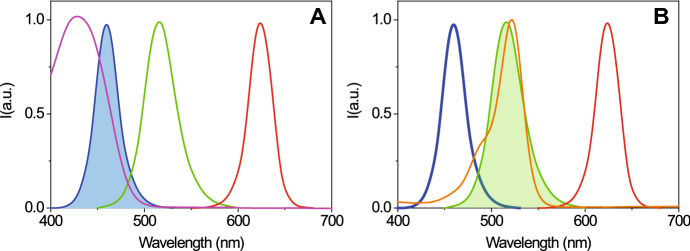
Typical normalized emission curves from an RGB LED array (red, green, and blue curves) overlapped to the absorption spectra of curcumin in ethanol (**a**, magenta) and eosin in PBS buffer (**b**, orange). The RGB component used in combination with the PS is highlighted by the shaded area

Taking into consideration the overlap between the absorption spectrum of the PS and the emission from the light source it is possible to provide a correct estimate of the number of absorbed photons [351]. This is exemplified in Fig. 21 for the RGB LED matrix output spectra and the absorption spectra of curcumin (panel A) and eosin (panel B).

A qualitative expression that provides an estimate of the number of absorbed photons per second by the PS is:

$$N_{{{\text{abs}}}} = \int {\left( {1 - 10^{ - \varepsilon \left( \lambda \right)cl} } \right)P_{{{\text{em}}}} \left( \lambda \right)\frac{\lambda }{{hc_{0} }}{\text{d}}\lambda } ,$$

where *ε*(*λ*) [M^−1^ cm^−1^] is the molar exctinction coefficient at the wavelength *λ*, *P*_em_(*λ*) [mW/nm] is the spectral radiant power of the light source, *c* [M] is the concentration of the PS, *l* is the thickness of the solution, *λ* is the wavelength in nm, *c*_0_ is the speed of light in a vacuum, and *h* is Planck’s constant [352]. Normalization of the photosensitization data to the above parameter allows to compare biological efficacy of distinct PSs independent of their photo-physical absorption characteristics [352].

The light sources employed in photodynamic therapy have been recently reviewed [353], where general aspects of light dosimetry in relation to the specific application have been also discussed [354]. There are three main categories of light sources used in photodynamic treatments: lasers, LEDs, and lamps. Lasers of interest to photodynamic applications are mostly diode lasers, and provide highly monochromatic, collimated powerful beams that are easily coupled to optical fibers. They are widely used for superficial and interstitial photodynamic applications. LEDs generally have a lower output power compared to a diode laser, but they have lower cost and can be assembled in a variety of geometries. Typical output warrants light fluence exceeding 20 mW/cm^2^ in the selected spectral range. Lamps sources used in photodynamic applications include fluorescent, incandescent, metal halide, xenon arc, and sodium arc lamps. Although in principle, they can be coupled to optical fibers, the inherent coupling losses are high and therefore their use is mostly restricted to superficial applications [353, 355–357].

Modeling the light dose that is delivered to the tissue interested by the photodynamic treatment is a complex task that has been discussed [358]. Optimization of the in vivo therapeutic efficacy needs a comprehensive study of the PDI action spectrum that depends on both the PS absorption and the tissue optical properties [359]. In general, living tissues absorb and scatter incident visible light, to an extent that is inversely proportional to the wavelength of the incoming radiation. In addition, different tissues have different optical properties, including absorption and scattering.

**Fig. 22:**
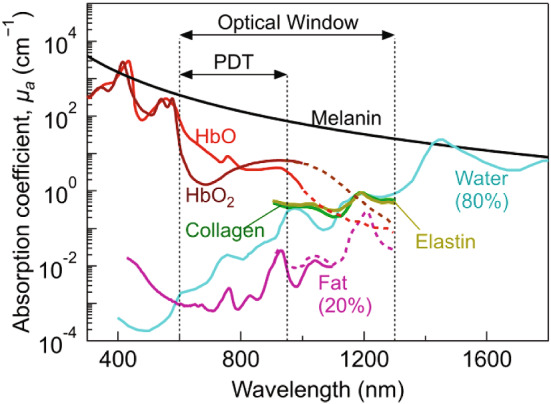
Absorption coefficient as a function of wavelength for several tissue constituents. Reproduced from Algorri et al. [360]

In particular, absorption of tissues receives contribution from their molecular components. Figure 22 compares the coefficients of tissue constituents hemoglobin, water, fat, elastin, collagen and melanin [360]. Melanin and hemoglobin (Hb) have the highest absorption coefficient (*μ*_a_) for wavelengths below 1 μm and dominate the absorption in this region. In spite *μ*_a_ of melanin being high, it is typically localized at low concentration in specific regions (e.g., skin), thus its absorption is not relevant in most tissues. For wavelengths above 1 μm, water dominates absorption. Further tissue dependent contributions come from fat, collagen, elastin. In view of the *μ*_a_ of main tissue constituents, an optical transmission window exists between 600 and 1300 nm. The optimal wavelength range for PDI is further limited by the need to achieve efficient ^1^O_2_ sensitization from the triplet excited state of the PS, which requires the use of wavelengths shorter than 800 nm [361, 362] and leads to an optimal therapeutic window between 600 and 800 nm [353].

Photodynamic treatments are restricted to biological targets that are optically accessible, either directly (e.g. skin or superficial wounds infections) or endoscopically through specialized optical fibers equipped with diffusing tips or microlenses, depending on the geometry of the tissue to be treated. PDI light delivery devices are classified as non-contact, contact, and interstitial [353].

## Conclusions

Antimicrobial photodynamic treatments are gaining increasing attention as an effective alternative approach to antibiotics or antivirals for localized infections, in consideration that antibiotic-resistant bacteria and emerging viral infections are threatening modern health care worldwide.

Although the first demonstration of the method against bacteria dates to the early twentieth century, and a number of investigations have explored the potential of photodynamic treatment in the battle against pathogens, development of PDI into a clinically accepted therapy is still moving the first steps. In conclusion, we wish to make a few comments on a selection of remaining critical issues that, if solved, may contribute to improve PDI.

Exploiting specific interactions with molecular components at the bacterial wall, nano- and biotechnology approaches have devised nanoparticles and supramolecular systems endowed with remarkable photoantimicrobial properties. However, in comparison with tumor PDT, where several receptors on tumor cells have been successfully targeted, e.g., with antibodies or engineered peptides, targeting of bacteria and viruses is a more difficult task to achieve. Although several strategies have been explored, the choice is so far quite limited.

On the other hand, in terms of the development of photoactive molecules, the research has been quite productive over the years. While the discovery of conventional antibiotics during the last 20 years has been quite insufficient, several classes of photosensitizing molecules have been proposed, either synthetic or of natural origin. Although many of them have shown encouraging results for in vitro studies on planktonic cultures, much less has been done on bacterial biofilms, where the development of suitable protocols may be necessary to overcome the current limitations in the application of the treatment.

Several aspects concerning the photophysics of PS molecules, especially when present in multiple copies on supramolecular systems or nanoparticles, are still challenging the current research. For instance, interactions between photoactive molecules on the supramolecular structures often results in quenching of excited states, with a net decrease in photosensitization efficiency and fluorescence quantum yield. Molecular design and spectroscopic investigations in this direction are needed to address this issue.

Monitoring real time production of ROS and their effects on biological structures is still a formidable task, due to the small size of pathogens (from 1 μm for bacteria to a few tens nanometers for small viral particles) that requires the use of imaging techniques with nm resolution, and the need to develop suitable molecular reporters (e.g. fluorescent probes) of the reactive species. Although singlet oxygen itself emits phosphorescence, its spectral range and the extremely low quantum yield render the use of this probe quite problematic for cellular studies.

Finally, identification of the main target structures that are damaged or destroyed by the photodynamic action is a crucial step for evaluating the performance of the PS and the possible development of resistance. Given the small size of bacteria and viruses that limits the use of conventional fluorescence microscopy, mostly biochemical methods or electron microscopy have been used to this purpose. The advent of fluorescence microscopy methods with subdiffraction resolution has opened new perspectives that are still almost completely unexplored, and may gain access to previously unavailable correlation between PS localization, microbial structures, and photoinduced damages. Moreover, important in situ kinetic information on PS uptake, ROS production and structural damage may become available. The engineering of probes with suitable in situ spectral properties will be needed to obtain multiple readouts with minimal interference. Thus, spectroscopic studies of these photoactive compounds when bound to the target biological systems are needed to ensure that photosensitization is preserved, and that the photophysical properties exploited by super-resolution fluorescence microscopies are kept.
